# Global review of bull shark *Carcharhinus leucas* (Valenciennes, 1839) museum specimens: summary of records, geographic analysis, and assessment of their scientific value

**DOI:** 10.3897/BDJ.14.e205769

**Published:** 2026-11-02

**Authors:** Mariana Haueisen, Luiz Fernando Salvador-Jr., Peter Gausmann

**Affiliations:** 1 Museum of Zoology, University of São Paulo, São Paulo, Brazil Museum of Zoology, University of São Paulo São Paulo Brazil; 2 Neotropical Research - Grupo de Estudo para a Conservação da Fauna Neotropical, Belo Horizonte, Brazil Neotropical Research - Grupo de Estudo para a Conservação da Fauna Neotropical Belo Horizonte Brazil; 3 Geographisches Institut, Ruhr-Universität Bochum, Bochum, Germany Geographisches Institut, Ruhr-Universität Bochum Bochum Germany

**Keywords:** Chondrichthyes, Elasmobranchii, Carcharhinidae, cosmopolitan species, data gaps, distribution, ichthyological collections, natural history, review

## Abstract

Background

Museum specimens provide critical data for taxonomy, systematics, biogeography, and conservation. Well-preserved vouchers are essential for documenting both historical and contemporary distributional records, supporting a better understanding on species distribution in a world undergoing constant environmental changes. Regarding the euryhaline bull shark, *Carcharhinus leucas*, voucher specimens reliably confirm occurrences in areas where the species' status was previously unclear and clarify the identity of morphologically similar shark species found in freshwater environments. This study aims to provide a synthesis of *C. leucas* voucher specimens, assessing their scientific significance and identifying priority regions for future sampling to address knowledge gaps in the occurrence and distribution of the bull shark.

New information

This study is one of the few available comprehensive global reviews of museum specimens for an extant shark species. By reviewing public databases and the primary literature, we collated information on 964 *Carcharhinus leucas* voucher specimens housed in ichthyological collections worldwide, of which 830 (86.1%) were associated with spatial metadata that allowed for geolocating of specimens. Among the specimens with spatial information, 6.3% (n = 52) were collected in freshwater, 49.0% (n = 407) in brackish estuarine habitats, 43.9% (n = 364) in marine environments, and 0.8% (n = 7) lacked precise locality so were not attributable to one of these categories. Our spatial analysis revealed several regions where collection efforts for *C. leucas* should be prioritized, including the Pacific and Atlantic coasts of South America, the Southwest Atlantic region of Africa, and the entirety of the Indian Ocean coast. The examination of this material confirmed the species' presence in locations where its occurrence was previously uncertain, and pre-dated its known occurrence in the Brazilian Amazon. Furthermore, our investigation revealed that some vouchers are imprecisely geolocated, either by the original collectors or during subsequent curation, highlighting the need for optimized spatial precision and caution when utilizing these data for scientific studies.

Key findings of our study new to science:

1. A total of 964 *Carcharhinus leucas* vouchers (excluding fossils and genetic samples) were identified across global natural history collections.

2. Critical sampling gaps were identified in the Global South, particularly along the Atlantic and Pacific coasts of Central and South America, the Atlantic and Indian Ocean coasts of Africa, and vast regions of Asia (Indian and West Pacific).

3. Distributional data do not support the presence of *C. leucas* north of Bahía Magdalena (southern Baja California, Mexico); records north of this limit remain unconfirmed.

4. Direct examination of material verified the species' presence in Samoa (Polynesia, Southwest Pacific Ocean) and the Azores Archipelago (Portugal, North Central Atlantic Ocean).

5. Three previously undocumented estuarine/riverine records were identified for Puerto Rico (n = 2) and Colombia (n = 1).

6. The first record of *C. leucas* in the Amazon River (Belém, Pará, Brazil) was pre-dated to 1903, ten years earlier than previously reported in the scientific literature (1913). Simultaneously, this specimen also represents the first documented record of the species for Brazil.

7. Historical vouchers from riverine locations currently inaccessible to migratory fish (due to anthropogenic barriers) provide empirical evidence of habitat degradation and loss of fish biodiversity.

## Introduction

Planet Earth shelters remarkable fish diversity, encompassing almost 37,600 valid vivant species (Fricke et al. 2026). However, due to human population growth and the consequent contemporary society' s demands, this richness has largely suffered from habitat loss, pollution, and overexploitation (Sala and Knowlton 2006, Sayer et al. 2025, Vardakas et al. 2025). As a consequence, the world has experienced a global fish biodiversity crisis, leading to sharp stock declines and even species extinctions in the wild (Lanjewar et al. 2024, Sayer et al. 2025, Vardakas et al. 2025). In addition to the direct effects of human actions, climate change also poses a serious threat to life in aquatic environments, potentially altering life cycles, ecological interactions, and distribution of many fish species, among other disturbances (Nagelkerken et al. 2023, Kawade et al. 2025).

In this current scenario, sharks are among the most imperilled groups, with one-third of all extant species threatened with extinction (Dulvy et al. 2021, Jabado et al. 2025). Due to their late age at maturity and low fecundity, which result in low reproductive output and slow population recovery, sharks are suffering from unregulated fisheries, habitat loss, and human-driven changes in food webs (Baum et al. 2003, Gallagher et al. 2014, Dulvy et al. 2021, Pacoureau et al. 2021). Moreover, the numbers of large coastal predatory sharks are also declining due to persecution and anti-shark measures (e.g., culling, shark meshing), mainly in regions where incidents involving humans have been recorded (Dudley 1997, Dudley and Simpfendorfer 2006, McCagh et al. 2015).

The bull shark *Carcharhinus leucas* (Valenciennes, 1839) is a large, coastal apex predator with a cosmopolitan distribution, attaining lengths up to 400 cm total length (TL) and weights of ~600 kg (McCord and Lamberth 2009). It is predominantly associated with shallow waters of the continental shelf (0-30 m water depth), occurring in tropical, subtropical, and warm-temperate regions of the world (Compagno 1984, Ebert et al. 2021, Gausmann 2021).

Unlike the vast majority of elasmobranchs (sharks, skates, and rays), the euryhaline bull shark is able to maintain osmotic and ionic homeostasis across a wide range of salinities (Pillans and Franklin 2004, Pillans et al. 2005, Reilly et al. 2011). Mature females typically give birth in estuaries, and juveniles remain for years in associated rivers and lakes before returning to the sea (Gausmann 2021). This behaviour of entering low-salinity habitats has evolved to reduce offspring mortality via lower predation risk provided by low-salinity environments (Heupel and Simpfendorfer 2011). As such, in large streams, bull sharks can swim thousands of kilometres upstream from river mouths and persist in freshwater habitats for several years during their early life stages (Gausmann 2021).

As recorded for many shark species, *C. leucas* is captured as a target species and as bycatch throughout its range (Rigby et al. 2021). Beyond unsustainable fisheries, the species also suffers from the loss and degradation of critical inshore habitats, including riverine, lacustrine, and estuarine nurseries upon which it essentially relies (Cutler et al. 2020, Rigby et al. 2021, Gausmann et al. 2025). Consequently, *C. leucas* is currently listed globally as Vulnerable on the IUCN Red List of Threatened Species (Rigby et al. 2021).

The bull shark was the subject of recent reviews dealing with its global distribution (Haueisen 2020, Gausmann 2021), which revealed substantial knowledge gaps regarding its known distribution and life-history traits. These knowledge gaps present barriers to successful conservation efforts targeting this species. However, based on advances in shark science, several aspects of the bull shark's complex biology and ecology have been unveiled in recent years (Gausmann 2024, Daly et al. 2025). These recent findings underscore how incomplete our understanding of its natural history remains, including physiology, life-cycle, and biogeography.

Biological collections play a fundamental role in the conservation of biodiversity, serving as permanent sources of information on taxonomy, systematics, biogeography, and evolutionary history (Suarez and Tsutsui 2004, Castillo-Figueroa 2018, Buckner et al. 2021). Museum collections constitute a primary source of information in biological sciences. As such, the collection of living material during field investigations and their subsequent deposition in scientific collections corresponds to good scientific practices and with a long-standing history (Castillo-Figueroa 2018, Rohwer et al. 2022). Although current efforts frequently prioritize the documentation of rare or morphologically similar species that are challenging to distinguish and unusual or remarkable occurrences in out-of-the-ordinary places, the collection of common and widely distributed species is also essential to ensure that these records are scientifically verifiable rather than merely anecdotal. Accurately labeled voucher specimens provide empirical evidence of a species' presence in a certain area at a well-defined time, thereby contributing to robust faunistic inventories at different spatial scales.

Beyond their scientific value for morphological studies and taxonomic revisions, voucher specimens enable genetic investigations, provided that DNA has not been damaged by preservative fluids used during long-term storage (Suarez and Tsutsui 2004, Castillo-Figueroa 2018, Meineke et al. 2018). They even allow for the extraction of ancient DNA from collected shark specimens (Straube et al. 2021a, Straube et al. 2021b). As such, museum collections offer unique opportunities to investigate collected material through new techniques, including historical DNA analysis and museomics, which enhance our understanding of biodiversity and advance taxonomic studies (Palandačić et al. 2024, Schmid et al. 2025).

Moreover, voucher specimens may be the only evidence of historic occurrences of particular species in certain locations or regions where they are currently extirpated, thereby documenting their historical distribution (Paci et al. 2025). This also applies to elasmobranchs in certain areas where natural history collections have helped reconstruct their historical diversity (Agne et al. 2022, Crobe et al. 2026). Therefore, voucher specimens facilitate the establishment of connections between past and present through temporal comparisons, supporting the diagnoses of possible local extinctions and even their underlying causes. This makes them invaluable for reconstructing the species' historical ranges (Peixoto et al. 2019, Roberts and Jarić 2020, Castañeda-Rico et al. 2022).

In this context, museum fish collections serve as global biodiversity archives for elasmobranchs, offering extensive geographic coverage through specimens from all over the world (Thiel et al. 2009). Amidst accelerating environmental changes, the historical data within these collections enable comparative analyses of modifications in waterscapes, as well as spatiotemporal shifts in species distributions (Suarez and Tsutsui 2004, Meineke et al. 2018). The systematic investigation of shark museum holdings allows researchers to fill knowledge gaps regarding distribution and basic biological traits (Tsikliras and Dimarchopoulou 2021). Consequently, voucher specimens possess immense scientific value, playing a vital role in monitoring species declines and biodiversity loss while providing a foundation for robust global biodiversity assessments (Suarez and Tsutsui 2004, Castillo-Figueroa 2018, Meineke et al. 2018).

Nevertheless, preserving intact large-bodied sharks (> 200 cm TL) presents significant logistical challenges due to storage limitations. Consequently, in these cases, usually only specific structures are retained, such as jaws, fins, claspers, and/or skin. Thus, the preservation of whole specimens of large-bodied sharks is overwhelmingly biased toward embryos, neonates, young-of-the-year, and small juveniles that fit conveniently into standard preservation jars. As a result, immature specimens of sharks predominate in museum collections, creating an ontogenetic bias that precludes comprehensive morphometric examinations of adult material.

Whether entirely or partially, shark material deposited in institutional collections facilitates the clarification of ambiguous records and the identification of morphologically similar species through direct comparison. Regarding the bull shark, taxonomic confusion often arises with species that share overlapping habitats or similar appearances, such as the Ganges shark *Glyphis gangeticus* (Müller and Henle, 1839), other river sharks within the genus *Glyphis* (Agassiz, 1843), and the pigeye shark *Carcharhinus amboinensis* (Müller and Henle, 1839). Misidentifications of *C. leucas* with these taxa have occurred numerous times in the past, especially concerning records of "river sharks" in freshwater and brackish environments (e.g., Compagno et al. 2010, White and Last 2012). For instance, Compagno et al. (2005) highlighted challenges in assigning historical Philippine freshwater elasmobranch records to specific species. This taxonomic uncertainty could have been avoided through the collection of voucher specimens for definitive examination. Moreover, not all freshwater carcharhinid records represent *C. leucas*, as the ability to inhabit freshwater is not restricted to this species, since other members of the genus *Carcharhinus* also occupy riverine habitats (Iqbal et al. 2019). Thus, Shell et al. (2023) emphasized that the verification of riverine elasmobranchs should ideally be based on voucher specimens. This practice would prevent taxonomic confusions arising from the morphological similarity between many *Carcharhinus* species (De Silva 2014).

Given its sympatry with other carcharhinids, voucher specimens of *C. leucas* are especially relevant to science, as many aspects of the species' genetics, taxonomy, life history, and distribution remain unresolved. For example, divergent anatomical and genetic data from populations across its range suggest that *C. leucas* may represent a species complex (Garrick 1982, Naylor et al. 2012, Pirog et al. 2019, Devloo‐Delva et al. 2023, Postaire et al. 2024). Considering its unique life-history traits, Vulnerable conservation status, geographical knowledge gaps, and taxonomic uncertainty, it is imperative to better understand structure and differentiation among bull shark populations originating from different ocean basins from a global perspective. Future analyses of museum material could serve as a basis for clarifying the taxonomic status of *C. leucas* populations.

Thus, our study aims to evaluate and map *C. leucas* voucher specimens from ichthyological collections worldwide to provide a spatial analysis and a comprehensive list of specimens, in order to address the following key research questions:

1. What is the origin of collected *C. leucas* material, regarding freshwater, brackish, and marine environments within global collections?
2. What is the geographic coverage of *C. leucas* voucher specimens, and how can this information reveal data-poor regions that represent significant gaps throughout the species' cosmopolitan distribution?
3. What are the scientific and conservation implications of collecting, curating, and investigating voucher specimens of threatened shark species?
4. What are the recommendations for the curation of whole shark specimens or their parts to ensure accurate species identification?

To address these research questions, we compiled and analyzed data on *C. leucas* voucher specimens available in global ichthyological collections and online databases. Our study provides a comprehensive inventory of *C. leucas* vouchers and their institutional distribution, serving as a resource to assist the scientific community in order to locate and access museum specimens for future investigations. This dataset delineated the geographic coverage of available material and served as a basis for spatial analyses of specimen provenance, highlighting significant gaps across several geographic regions. The records were also used to investigate morphological and taxonomic characters, providing clarification in instances where the species identity was previously uncertain. Finally, the compiled data provide an assessment of the current state of museum records for this euryhaline predator.

## Material and methods

To analyse the spatial pattern of globally distributed bull shark museum records, we carried out an extensive search for voucher specimens of *Carcharhinus leucas* deposited in ichthyological collections worldwide (Suppl. material 1). Our data acquisition encompassed public databases, literature records, and information from institutional collections obtained through direct communication with curators.

Regarding the public databases, we queried the Global Biodiversity Information Facility (GBIF – Global Biodiversity Information Facility 2025), Specieslink (CRIA 2025), Field Museum's online Zoological Collections Database (Field Museum 2025), FishBase (Froese and Pauly 2025), FishNet 2 (National Science Foundation 2017), Harvard Museum of Comparative Zoology (Harvard University 2026), Integrated Digitized Biocollections (iDigBio – Integrated Digitized Biocollections 2025), and Atlas of Living Australia (ALA – Atlas of Living Australia 2025), the latter also including the datasheet with records from the Northern Territory Ichthyological collection, Northern Territory Museum, Darwin (Dally 2023). All these datasheets provide access to information on collected specimens housed in various museums and institutions around the world.

Moreover, we requested information on several collections from the following ten museums and institutions for species verifications:

- American Museum of Natural History, Department of Ichthyology (New York City, USA);
- California Academy of Sciences, Department of Ichthyology (San Francisco, USA);
- Museu de Ciències Naturals de Barcelona, Department of Vertebrates (Barcelona, Spain);
- Museum für Naturkunde, Ichthyologische Sammlung (Berlin, Germany),
- Natural History Museum of Los Angeles County, Department of Ichthyology (Los Angeles, USA);
- Naturhistorisches Museum Wien, Zoologische Abteilung, Fischsammlung (Vienna, Austria);
- Scripps Institution of Oceanography, Marine Vertebrate Collection (San Diego, USA);
- Smithsonian Institution, Division of Fishes, Fish Collection (Suitland, USA);
- South African Institute for Aquatic Biodiversity (Grahamstown, South Africa);
- Texas A&M University, Fish Collection (College Station, USA).

We supplemented our dataset with records published in scientific literature (e.g., country checklists of elasmobranchs, morphometric studies) in which *C. leucas* voucher specimens were cited (Suppl. material 2). This integration facilitated cross-referencing of vouchers and provides a framework for future research by providing additional information on literature references.

The temporal scope of the collected voucher specimens spanned from 1845 to 2024. Although some specimens lacked formal collection dates, they were retained in the dataset ( Suppl. material 1). Regarding the scientific literature, the compiled records encompassed documents published between 1948 and 2026 (Suppl. material 2).

To standardize our dataset based on these records, Buckner et al. (2021) defined a voucher as a permanently preserved specimen that is maintained in an accessible collection. However, given the diverse array of record types for *C. leucas*, we first restricted our scope to collected material from living specimens by excluding all fossil records. Since *C. leucas* is an evolutionarily ancient taxon, with a stratigraphic record extending to the Miocene (Ebersole et al. 2017) and recent forms present since the Miocene/Pliocene (Latrubesse et al. 1997), fossil shark teeth of *C. leucas* have been reported from many regions across the world where the species no longer occurs due to continental drift and associated paleoclimatic shifts. Therefore, they do not contribute to our understanding of the species' contemporary distribution.

Moreover, since this study specifically focuses on physical voucher material, comprising whole specimens or anatomical parts, genetic samples were also excluded from the analysis, though these records are summarized in the supplementary data (Suppl. material 1). Consequently, only records cataloged as "Preserved Specimen" within public databases were retained for investigation, with the exception of a visual observation that was formally accessioned with a voucher number.

Following these exclusions, the remaining vouchers were categorized based on the availability and quality of their information into two main groups: (1) specimens with associated locality information; and (2) specimens lacking locality data ( Suppl. material 1).

Our search also included records for *C. leucas* labeled with its numerous synonyms historically used in different regions of the world (Garrick 1982, Pollerspöck 2011, Fricke et al. 2026):

- Carcharias (Eulamia) azureus Gilbert and Starks, 1904 (Tropical Eastern Pacific);
- *Carcharias spenceri* Ogilby, 1910 (East Australia);
- Carcharias (Prionodon) zambezensis Peters, 1852 (Southeast Africa);
- *Carcharhinus leucas leucas* Urist, 1962 (North America);
- *Carcharhinus leucas nicaraguensis* Urist, 1962 (Central America);
- Carcharhinus (Squalus) platyodon Jordan and Evermann, 1896 (North and Central America);
- *Carcharhinus vanrooyeni* Smith, 1958 (Southeast Africa);
- *Eulamia nicaraguensis* Gill in Gill and Bransford, 1877 (Central America);
- Galeolamna (Bogimba) bogimba Whitley, 1943 (East Australia);
- *Galeolamna greyi mckaili* Whitley, 1945 (West Australia);
- *Galeolamna mckaili* Whitley, 1951 (West Australia);
- *Prionodon platyodon* Poey, 1860 (Caribbean Sea);
- *Squalus obtusus* Poey, 1861 (Caribbean Sea).

Institutional and museum abbreviations primarily adhere to the standardized codes established by Sabaj (2020) (Suppl. material 3). For each voucher detected, we recorded the hosting institution, catalog number and associated metadata — including collection locality (country, region, and geographic coordinates), sampling date, collectors, and the number of specimens represented ( Suppl. material 1). To facilitate spatial analysis, each voucher was treated as a single occurrence record, regardless of the number of individuals it comprised. Vouchers from institutions with uncertain taxonomic reliability were generally excluded. However, in cases of noteworthy biogeographic significance, ambiguous records were subjected to individual verification to validate species identity and record accuracy. This was accomplished by requesting supplemental information, such as photographic material, from collection managers and curators to perform a definitive taxonomic confirmation.

Specimen mapping was conducted with maximum spatial accuracy, utilizing either precise geographical coordinates (original and unambiguous) or estimated locations derived from associated locality description (localities, regions, and administrative territories). Those records lacking precise original coordinates were manually georeferenced by verifying localities through Google Earth® and Google Maps®, thereby providing spatial approximations for the occurrence points. For vouchers whose locality description were regional in scope, approximate coordinates were determined based on further records within the same region that possessed exact coordinates. On the other hand, vouchers lacking sufficient information for reliable georeferencing were excluded from the spatial analysis, although these records remain documented in the comprehensive dataset (Suppl. material 1). All spatial data were processed and visualized using QGIS® software.

For habitat classification purposes, salinity characteristics for each voucher locality were identified through an extensive review of literature that contained environmental information about the specific locality ( Suppl. material 2). Based on these characteristics, we adopted (and adapted, where precise salinity data were unavailable) the framework synthesized by Elliott and McLusky (2002), categorizing environments into three groups based on salinity regimes and hydrological characteristics:

1. Fresh water (FW): This category included records from typical limnetic environments, such as inland lakes, artificial reservoirs, and river stretches upstream of significant saline intrusion and tidal influence;
2. Estuarine (E): Defined as the ecotone between the river and the sea, characterized by a measurable dilution of seawater with freshwater and tidal influence. This category includes estuaries, river mouths, river channels, lagoons, and bays exhibiting oligohaline to polyhaline regimes;
3. Sea water (SW): Comprises records from stable coastal and offshore environments characterized by true euhaline conditions.

## Results and discussion

### Origin and geographic distribution of voucher specimens 

**Figure 1.**
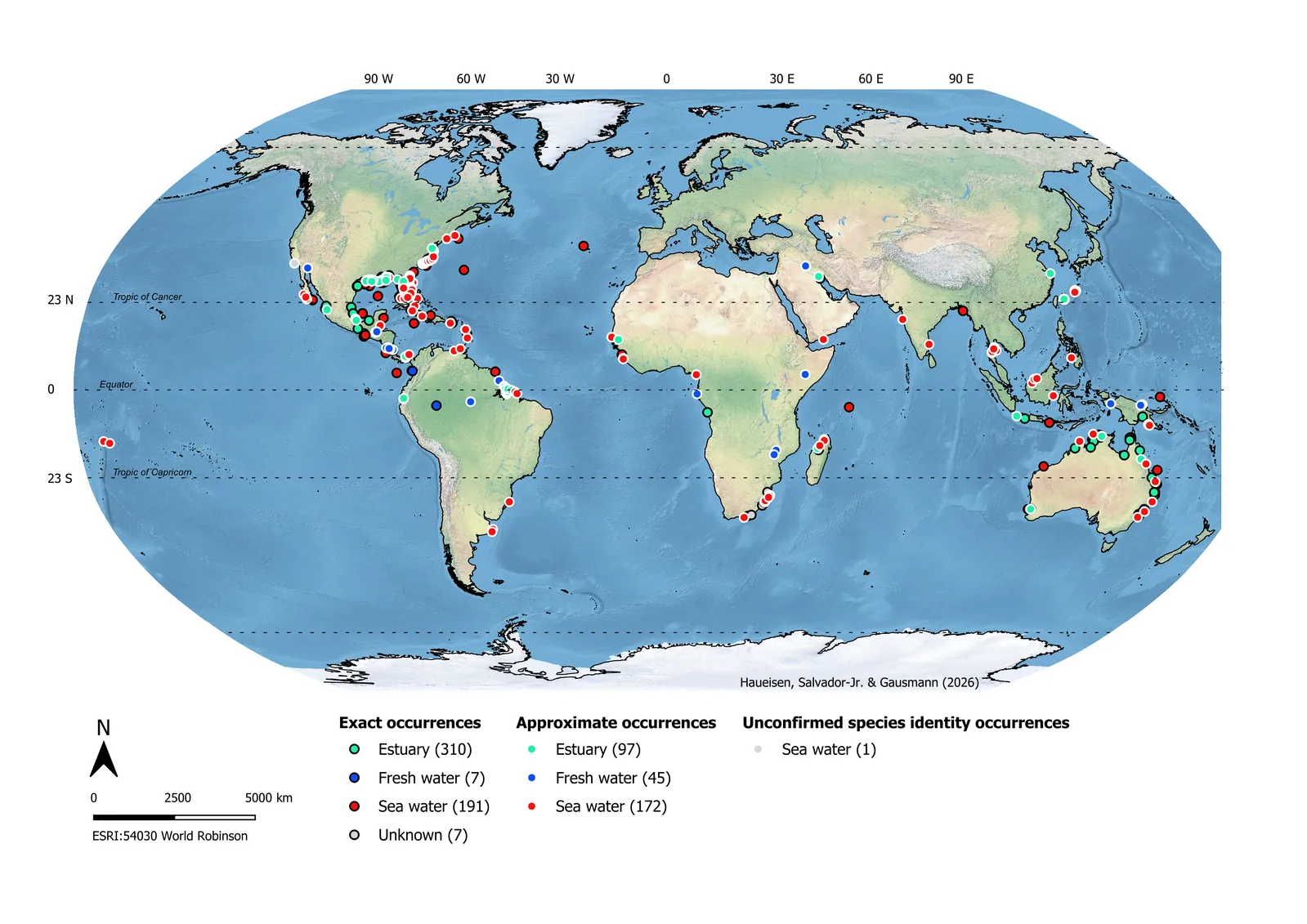
Global distribution of *Carcharhinus leucas* (recent taxon/fossils excluded) voucher specimens included in the present review.

Our global compilation of *Carcharhinus leucas* voucher specimens reveal a cosmopolitan distribution across all major ocean basins (Indian, Pacific, and Atlantic). However, this broad range is characterized by vast areas where voucher material of the bull shark remains absent (Fig. 1, Suppl. material 4). Although specimens originate from all aquatic environments and habitats utilized by the species — including inland freshwater rivers and lakes, river mouths/estuaries, and inshore/offshore marine waters — a pronounced geographical bias is evident.

Most vouchers come from developed nations, mainly the United States and Australia (Fig. 1). In contrast, significant portions of the developing world, such as West and East Africa, large sectors of Asia, and extensive stretches of the South American coastline lack representative voucher material. These gaps align with what we intend to address with our study, namely that large parts of the Global South lack voucher specimens due, in parts, to limited research funding. Moreover, our results corroborate the findings of Nakamura et al. (2025), who reported on an apparent knowledge split in biological collections between the Global North and the Global South.

**Figure 2.**
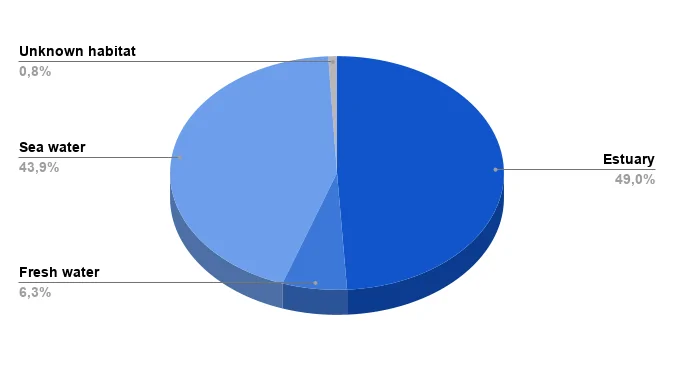
Assignment of collected material of *Carcharhinus leucas* (n = 830) linked to different habitat categories in percentages.

From the metadata associated with the 830 museum records that are attributed with spatial information, estuarine environments accounted for nearly half of the dataset (407 vouchers; 49.0%), underlining the strong affinity of this species for estuaries and river mouths. Marine settings followed with 364 specimens (43.9%), while freshwater occurrences represented 6.3% of the total (52 vouchers). A minor fraction (7 vouchers; 0.8%) could not be categorized due to incomplete geospatial information (Fig. 2, Suppl. material 5).

The results must be understood alongside the bull shark’s ontogenetic habitat shifts. Throughout their development, specimens of *C. leucas* utilize a broad array of habitats, ranging from low-salinity systems (rivers, lakes, estuaries) to coastal marine reefs and shallow shorelines (Carlson et al. 2010, Werry et al. 2011, Matich and Heithaus 2015). Newborns, young-of-the-year, and small juveniles are primarily found in freshwater rivers, lakes, and estuaries, while large subadults and adults live in the sea. This behavioral pattern is recognized as a strategic avoidance mechanism, enabling juveniles to escape both inter- and intraspecific predation by seeking refuge in low-salinity habitats away from the open ocean (Heupel and Simpfendorfer 2011). Anyway, the lower number records from freshwater reported here reflect the fact that although it is adapted to low salinity environments (Pillans and Franklin 2004, Pillans et al. 2005, Reilly et al. 2011), the bull shark is primarily a coastal species (Ebert et al. 2021).

### Freshwater records

The majority of *C. leucas* specimens from freshwater habitats were juveniles, but to a lesser extent also the subadult and adult age classes, ranging from 73.8 to 193.0 cm TL (Suppl. material 1). Consistent with the species' life history, these freshwater records corroborate the role of these environments as primary nurseries where neonates reside during their early life stages. Adults are rare in such habitats, with notable exceptions including a specimen collected in the Amazon River at Leticia, Colombia, in 1973 (CMNFI-1974-0095.1).

Material of *C. leucas* was collected from several streams associated with all major ocean basins worldwide, including the Amazon, the Zambezi, and the Tigris Rivers (Suppl. material 5). Moreover, *C. leucas* voucher specimens were derived from freshwater lakes in Central America, such as Lake Izabal and Lake Nicaragua, which were considered ancient nurseries of outstanding importance for this species in the Caribbean region (Gausmann et al. 2025).

Within the vast Amazon River system, only 35 records of *C. leucas* have been reported to date over a span of 110 years, which is relatively few considering the extensive nursery habitat provided by this basin (Gausmann et al. 2025). Of these 35 records, only one had been confirmed by a physical voucher specimen (CMNFI-1974-0095.1, preserved jaw), while another was documented via photographic material (MOVI-10179). In addition to these limited records, our identification of a previously unpublished specimen from Belém, housed in the collection of the Natural History Museum of Vienna (NMW-61418; see record no. 3 below), increases the overall number of *C. leucas* records from the Amazon River system to 36 and extends the number of museum specimens collected in this river with an additional specimen.

Furthermore, a previously unpublished freshwater record of *C. leucas* originates from a voucher specimen collected in 1966 on the Pacific slope of Colombia. This specimen was obtained inland near the village of Piliza (Chocó Department), representing a significant historical occurrence for the region's freshwater ichthyofauna. Although the voucher (NMNH-FISHES-45923; Suppl. material 1) was not labelled with a specific location name, its attributed coordinates derived from the metadata place this specimen within the Piliza River system, which drains into the Eastern Pacific Ocean. Records of *C. leucas* from the Pacific slope of the South American continent are scarce, yet they hold high biological importance for the species' reproduction in the Tropical Eastern Pacific (Gausmann 2021). This scarcity is largely attributed to the limited number of large riverine systems connected to the Pacific Ocean that provide suitable habitat for parturition and juvenile development. Consequently, the Piliza River warrants further investigation to assess its current status and potential function as a putative nursery area of *C. leucas*.

In the context of newly identified freshwater occurrences, our investigation revealed a record of *C. leucas* from Somalia's Juba River. This record was derived from a juvenile specimen collected far inland at Dolo (Dooloow), verified through the analysis of spatial information associated with this specific voucher (MSNG-CE36928). Although Gausmann (2021) previously referred to this occurrence and Garrick (1982) confirmed the species identity, our work highlights this voucher as a significant freshwater record that has remained overlooked by other scientific publications. Its inclusion here underscores the value of cryptic metadata hidden in museum collections, especially for well-catalogued specimens with key information not yet integrated into the scientific literature.

In remote and understudied regions that remain geographically inaccessible, museum specimens of *C. leucas* provide the primary evidence of the historical existence of sharks in inland waters. Between 1954 and 1955, the Dutch ichthyologist Marinus Boeseman collected four specimens of *C. leucas* (range: 73-148 cm TL) in the fresh waters of Lake Jamoer in West New Guinea (Papua), Indonesia (Boeseman 1964; RMNH-24611, 24697, 24698, 24699; Suppl. materials 1, 2). Due to the lack of field surveys following Boeseman's work, there are no subsequent confirmations of the presence of this species in Lake Jamoer until nowadays, which highlights that West New Guinea remains a data-deficient region regarding the contemporary occurrence of euryhaline elasmobranchs in freshwater. Furthermore, these four specimens are valuable for the natural history of *C. leucas* and the knowledge about the faunistic inventory of West New Guinea, as they represent the only available evidence of historical shark presence in Lake Jamoer. These historic records constitute the only documentation of *C. leucas* in the inland waters of West New Guinea to the present day and showcase the unpleasant paucity of information regarding freshwater elasmobranchs in this hardly accessible part of the world. Unfortunately, this temporal information gap does not allow a current evaluation of Lake Jamoer as a nursery for the bull shark (Gausmann 2021).

### Estuarine environments records

In comparison to other environments where *C. leucas* has been collected, sample locations within brackish water conditions comprise the largest proportion of the dataset (n = 407), with records derived from locations across multiple continents. These include the Chesapeake Bay (the largest estuary in the United States), the Shatt Al-Arab (bordering Iran and Iraq) — which discharges into the Persian Gulf — and the Swan River estuary in Western Australia (Suppl. material 5).

Our study revealed new, unpublished records of *C. leucas* from low-salinity environments that were not yet considered in the existing literature. These records include the Cibuco River (NMNH-FISH-112245) and the Apeadero River (MNHN-IC-A-9650), located on the north and south coasts of Puerto Rico, respectively (Suppl. material 5). Neither environment represents strictly freshwater conditions; rather, they correspond to brackish water at the original collection sites. While Neal et al. (2009) noted *C. leucas* in Puerto Rico's inland waters, specific localities were not provided. Because these two vouchers include precise spatial data, our investigation enabled accurate georeferencing and revealed two previously undocumented records from Puerto Rican inland brackish waters. Riverine and estuarine records of *C. leucas* from the Caribbean islands remain scarce, as nursery habitats are mainly suspected to occur along the continental coasts of Central and South America (Gausmann 2021).

### Sea water records

Voucher material of *C. leucas* was collected from sea water environments in all three major ocean basins — the Atlantic, Indian, and Pacific Oceans — including sub-basins such as the Caribbean Sea and the Gulf of Mexico (Suppl. material 5). However, there are large gaps along the coastlines of several continents. Vast stretches of the Southeast Pacific coast and the Southwest Atlantic coast of South America are, presently, data-deficient regions regarding extant museum material. The African coast, including both the Southeast Atlantic and the Western Indian Ocean, also lacks vouchers for considerable stretches of coastline and inshore habitats. The same applies to the Northern and Eastern Indian Ocean of Asia (Suppl. material 4).

Nevertheless, material from the Atlantic Ocean has unveiled and documented the presence of the bull shark at remarkable locations, specifically involving two extraordinary occurrences at the distributional range limits in the north-central and southwestern parts of the basin. The distributional limit of *C. leucas* in Argentine waters (Southwest Atlantic) is documented by two dried jaws (MACN-6873, MACN-6874), collected in 1976 off the coast of Buenos Aires Province (Chiaramonte 1998). This material currently remains the only known record of this species for Argentina until nowadays and represents the southernmost distributional limit in the Southwest Atlantic Ocean. The only record of *C. leucas* for the North Central Atlantic derives from the oceanic Azores Archipelago and is also documented by a set of a dried jaw (Gadig et al. 2006). Notably, the Azorean record represents the only record of a living *C. leucas* for the European continent and its adjacent territorial waters.

### Notable voucher specimens of scientific significance

In this section, we refer abbreviatedly and in an abstracted form to remarkable voucher specimens of *Carcharhinus leucas* that contain information of historical and/or biogeographical importance, with special emphasis on the distribution of this species at particular sites in freshwater, but also at locations where its occurrence was previously doubted. This section also includes specimens which are of special importance regarding the history of the scientific investigation of this species. More extendend and detailed information for each specimen can be found in the supplementary materials (Suppl. material 6).

**Figure 3.**
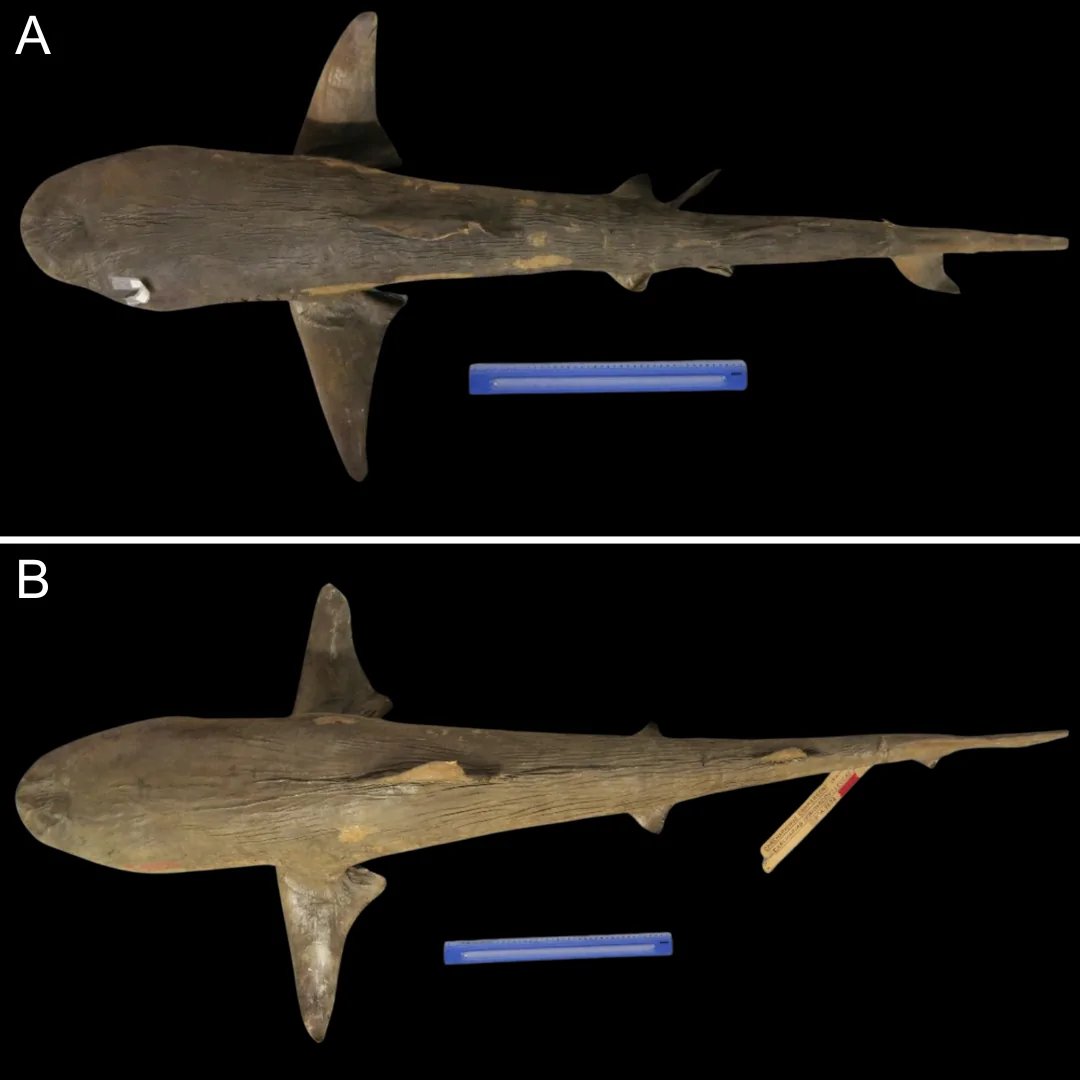
Dorsal view of the two taxidermied syntypes of Carcharias (Prionodon) leucas housed in the Muséum national d'Histoire naturelle (MNHN, Paris). **A** MNHN-IC-A-9650 (adult male, 160.0 cm TL, Antilles). **B** MNHN-IC-A-9652 (female, 186.9 cm TL, Antilles) (Reproduced with permission from Jonathan Pfliger/MNHN, under a CC BY 4.0 license).

**1. MNHN-IC-A-9650 – 160 cm TL, adult, ♂ and MNHN-IC-A-9652 – 186.9 cm TL, ♀, both from the Antilles (Fig. 3)**

• The two remaining type specimens of Carcharias (Prionodon) leucas.

• Two stuffed specimens in good condition (two further type specimens apparently lost during the Second World War).

• Collected in the Antilles (Caribbean Sea) by the French zoologist Achille Valenciennes who made the species description in 1839 (published in Müller and Henle 1841).

• The voucher specimen MNHN-IC-A-9650 (Fig. 3A) represents a mature male, as indicated by its fully calcified claspers, a character previously noted by Garrick (1982). This is one of the smallest mature male *C. leucas* on record. Additionally, the presence of other small, mature males in the Caribbean Sea (e.g., NMNH-120372) indicates that this population is morphologically distinct from other populations.

**Figure 4.**
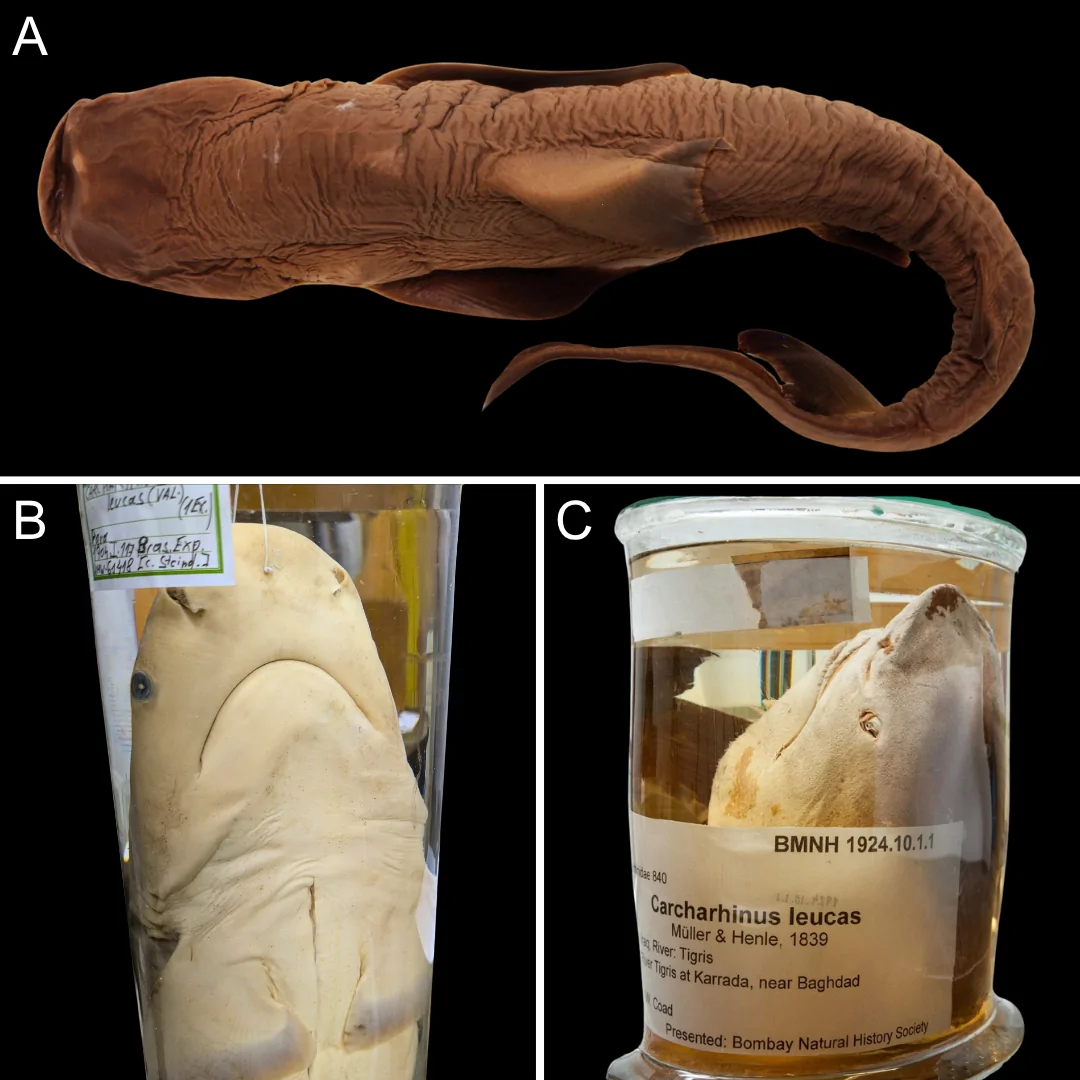
Voucher specimens of *Carcharhinus leucas* from inland waters. **A** Dorsal view of the voucher specimen (ISZZ-4468, ♂, 76 cm TL, holotype of Carcharias (Prionodon) zambezensis Peters, 1852, collected in 1845 in the Zambezi River at Tete, Mozambique, by W. Peters. This specimen as reported by Peters (1852), Peters (1868) represents the earliest globally verified freshwater record of *C. leucas* (Reproduced with permission from the Museum für Naturkunde Berlin, under a CC BY 4.0 license). **B** Specimen (NMW-61418, 66 cm TL) collected in the Amazon River at Belém (Pará State, Brazil) in 1903 by F. Steindachner. Housed in the Naturhistorisches Museum Wien (NMW), this voucher represents the first valid scientific record of the species from the Amazon River and the earliest documented occurrence for Brazil (Reproduced with permission from Peter Gausmann, under a CC BY 4.0 license). **C** Specimen (NHMUK-ZOO-1924.10.1.1, 124.4 cm TL, head only) collected in the Tigris River, Iraq, at Karrada near Baghdad in 1924. This record provides evidence of the historical use of the upper Tigris River, no longer accessible to this species due to riverine obstructions (Reproduced with permission from the Natural History Museum, London, under a CC BY 4.0 license).

**2. ISZZ-4468 – 76 cm TL, ♂, Zambezi River at Tete, Mozambique (Fig. 4A)**

• First verified freshwater record of *Carcharhinus leucas* to the scientific world.

• Collected in 1845 from the Zambezi River (Tete, Mozambique), 418 km upstream from the coast.

• Holotype of Carcharias (Prionodon) zambezensis Peters, 1852 (later revised as *Carcharhinus leucas* by John Garrick.

**3. NMW-61418 – 66 cm TL, ♂, Amazon River at Belém, Pará State, Brazil (**Fig. 4**B)**

• Collected in 1903 along the Amazon River at Belém (Pará State, Brazil) by the Austrian ichthyologist Franz Steindachner.

• First scientific record of the occurrence of *Carcharhinus leucas* in the Amazon River.

• First scientific record of *Carcharhinus leucas* for the country of Brazil.

**4. NHMUK-ZOO-1874.4.28.9 (BMNH-1874.4.28.9) and NHMUK-ZOO-1924.10.1.1 (BMNH-1924.10.1.1) (**Fig. 4**C), Tigris River near Bagdad, Iraq**

• Two voucher specimens collected in 1874 (NHMUK-ZOO-1874.4.28.9) and 1924 (NHMUK-ZOO-1924.10.1.1) in the Tigris River (Baghdad, Iraq), ~850 km upstream from the sea (Arabian/Persian Gulf).

• Both specimens constitute historical evidence of the extensive long-distance inland migrations and the use of the upper stretches of the Tigris River by juveniles of *Carcharhinus leucas*, areas that are currently inaccessible due to anthropogenic river barriers.

• Both specimens highlight the historical role of the Euphrates-Tigris-Karun-Shatt Al-Arab system (Iraq/Iran) as a critical nursery area for the species within the Arabian/Persian Gulf.

**5. ZSI-10250 – 65 cm TL, newborn, ♂, Hooghly River, India**

• Collected from India's Hooghly River in 1867 by John Anderson and validated by Compagno (1984), providing concrete evidence of the species' historical presence in the system.

• Particularly significant because the Hooghly River is the type locality of *Glyphis gangeticus*, a system where both species share a sympatric distribution (Compagno 1984).

**Figure 5.**
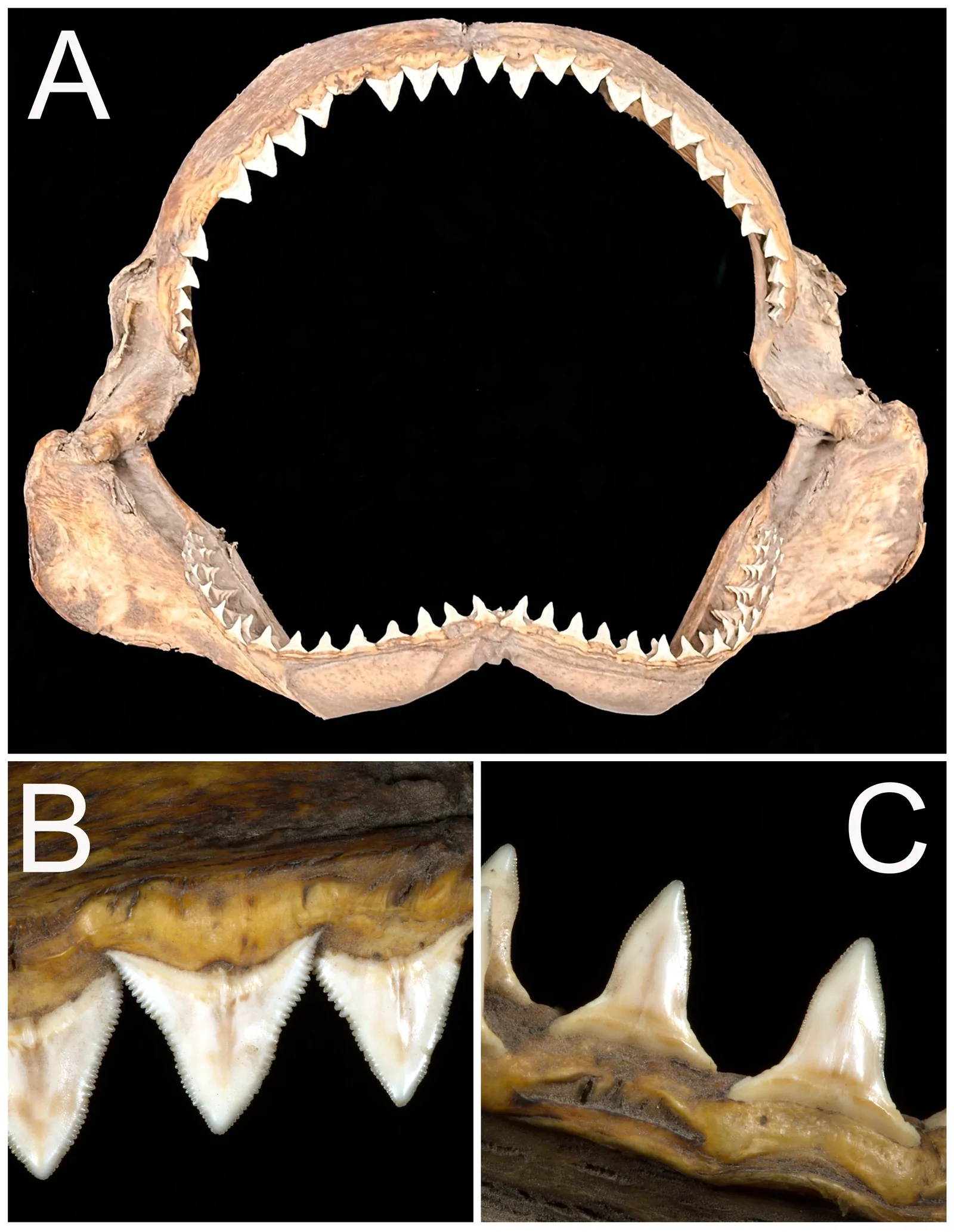
Jaw of the Samoan specimen of *Carcharhinus leucas* (CAS-ICH-55483). **A** Frontal view of the jaw; **B** close-up of the upper jaw dentition; **C** close-up of the lower jaw dentition (Reproduced with permission from the California Academy of Sciences, under a CC BY 4.0 license).

**6. CAS-ICH-55483 – Preserved specimen, jaws only, Samoa (Fig. 5)**

• This voucher specimen verifiably proves the presence of *Carcharhinus leucas* in Samoa where its presence was doubted.

**Figure 6.**
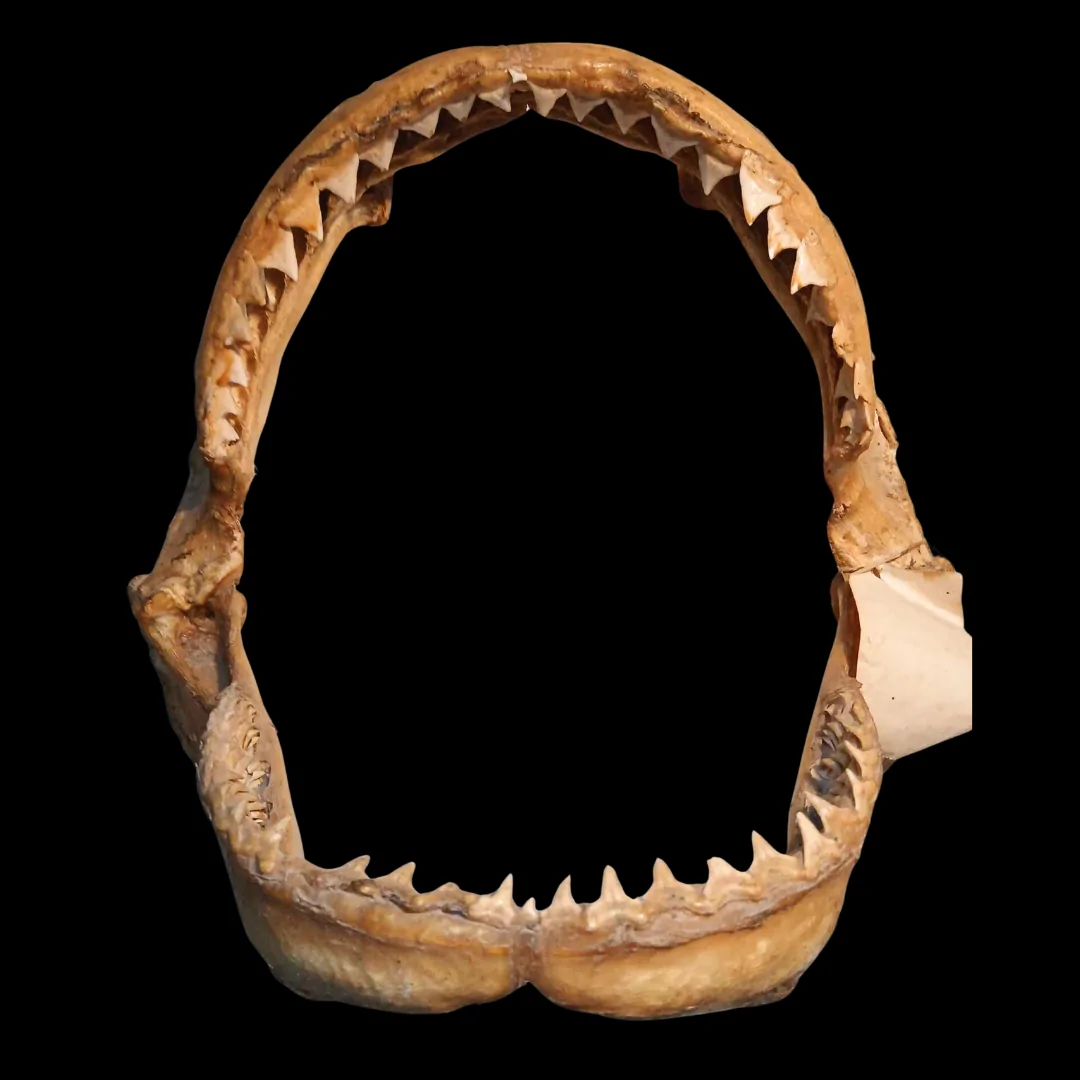
Jaw of the *Carcharhinus leucas* specimen (JPB, uncatalogued, ~250-270 cm TL, ♀) caught off the Azores Archipelago, Portugal, in 1993 (Reproduced with permission from João Pedro da Silva Ramos Barreiros, under a CC BY 4.0 license).

**7. JPB (João Pedro Barreiros – private collection; uncatalogued) - jaw from a specimen from the Azores Archipelago, Portugal, estimated 250-270 cm TL, ♀ (Fig. 6)**

• Jaws obtained from an adult female specimen of *Carcharhinus leucas* that was caught by Azorean fishers in 1993.

• First and only record of *C. leucas* for this oceanic archipelago, for Portugal, and for entire Europe.

• Possible indicator for transoceanic movements of *C. leucas* across the Atlantic Ocean and for ocean basin-wide connectivity.

**8. Specimens with distributional data for the Pacific Ocean north of Bahía Magdalena, southern Baja California, Mexico**

The following vouchers (8.1. to 8.6.) refer to specimens for which a collection site north of Bahía Magdalena was provided in the accompanying data, the northern limit of *Carcharhinus leucas* as reported by literature references.

**8.1. CAS-SU(ICH)-40091 – Preserved teeth**

• Collected in 1942 off Anacapa Island, Ventura County, California, USA (34.00° N, 119.40° W), from a 310 cm TL carcharhinid shark originally identified as *Carcharhinus azureus* (currently considered a synonym of *C. leucas*).

• Voucher apparently lost; preliminary assessed by the authors as unconfirmed.

**Figure 7.**
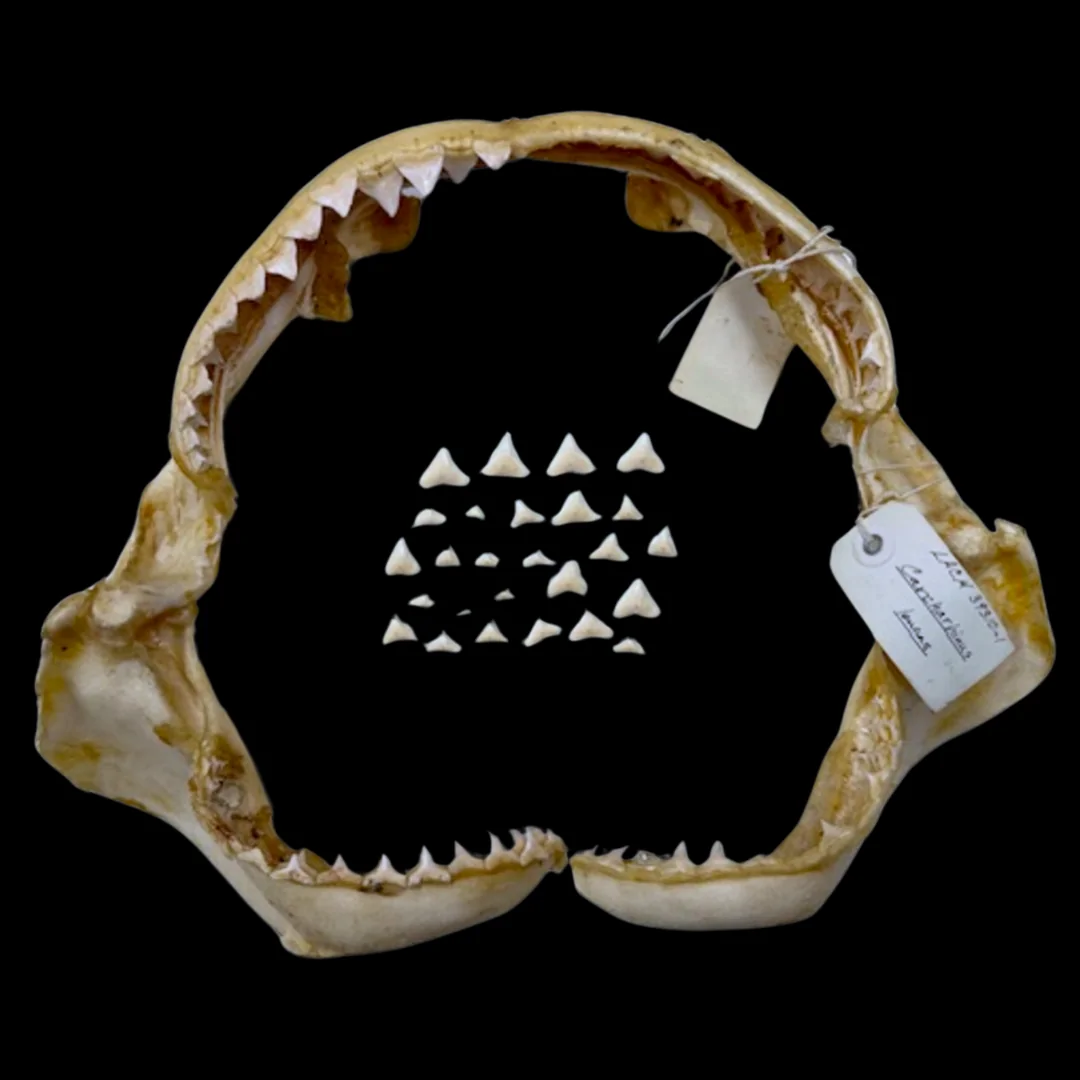
Jaw and associated dentition of voucher specimen LACM-Fish-39310-1 (western Baja California, Mexico; Northeast Pacific Ocean), confirmed as *Carcharhinus leucas* (Reproduced with permission from the Natural History Museum of Los Angeles County, under a CC BY 4.0 license).

**8.2. LACM-Fish-39310-1 (Fig. 7)**

• Voucher specimen comprises a preserved jaw and associated tooth set.

• The examination of photographic evidence (Fig. 7) supports the original species identification as *C. leucas*.

• However, the collector omitted the exact locality data, making georeferencing impossible due to the absence of precise spatial coordinates (and thus providing no evidence of occurrence north of Bahía Magdalena).

**8.3. MCNB-DelMoral-2015-842 and MCNB-DelMoral-2015-843**

• These two voucher specimens do not represent autonomous vouchers. Instead, they refer to specimens housed in other collections, specifically SIO-54-216 (MCNB-DelMoral-2015-842) and SIO-47-67 (MCNB-DelMoral-2015-843).

• As such, they don't contribute to the clarification if *C. leucas* occur north of Bahía Magdalena.

**8.4. SIO-47-67**

• Collected in September 1947 by a local fisherman and labeled with the locality data: "Baja California; N of San Felipe; Mar de Cortés" during the storage in the museum.

• Based on rather vague geospatial information, precise georeferencing of this specimen is impossible.

**8.5. SIO-54-216**

• This record represents a 1954 visual observation of a putative *Carcharhinus leucas* specimen (~244 cm TL) from Guadalupe Island, Mexico.

• Due to these diagnostic challenges and the presence of resembling congeners near Guadalupe Island, e.g., *C. obscurus*, this visual record was excluded from the mapping analysis.

**8.6. TCWC-Ichthyology-15531.01**

• This voucher is recorded from Bahía de Los Angeles, Gulf of California, Mexico (28.97° N, 113.48° W) and comprises only preserved claspers, which were likely already dissected by the curator.

• Because claspers among *Carcharhinus* congeners are very similar in appearance and lack distinctive diagnostic features, a reliable species identification was impossible.

**8.7. Summary of investigation and findings**

• Our investigation of the material listed above (8.1 to 8.6) do not support an occurrence of *Carcharhinus leucas* along the Pacific coast of Baja California north of Bahía Magdalena.

• As such, Bahía Magdalena remains the northern distributional limit of *C. leucas* at the Californian coast of the Northeast Pacific Ocean, as previous reported by the literature.

• The occurrence of *C. leucas* in US Californian waters is unsustained.

### Further results and discussion

**Figure 8.**
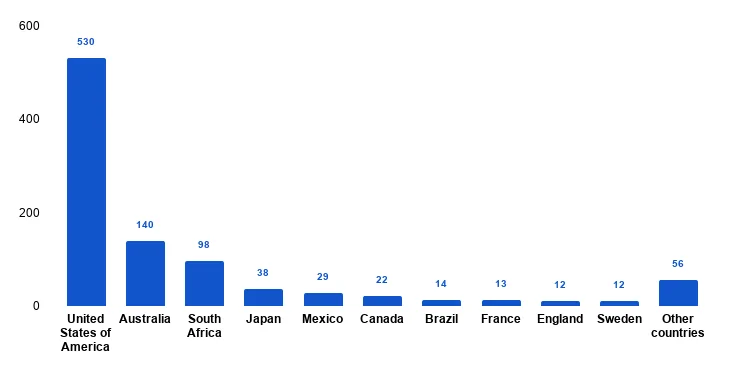
Frequency of the total number of *Carcharhinus leucas* voucher specimens (n = 964) categorized by the country of the holding institutions (n = 88; Suppl. material 1).

**Figure 9.**
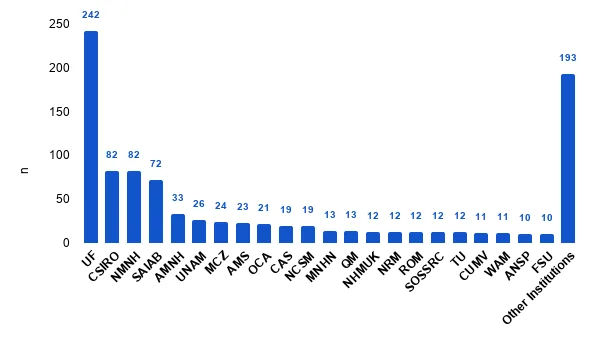
Distribution and absolute numbers of *Carcharhinus leucas* voucher specimens (n = 964) across museum institutions (n = 88). The chart highlights the 22 institutions holding 10 or more voucher specimens each.

**Figure 10.**
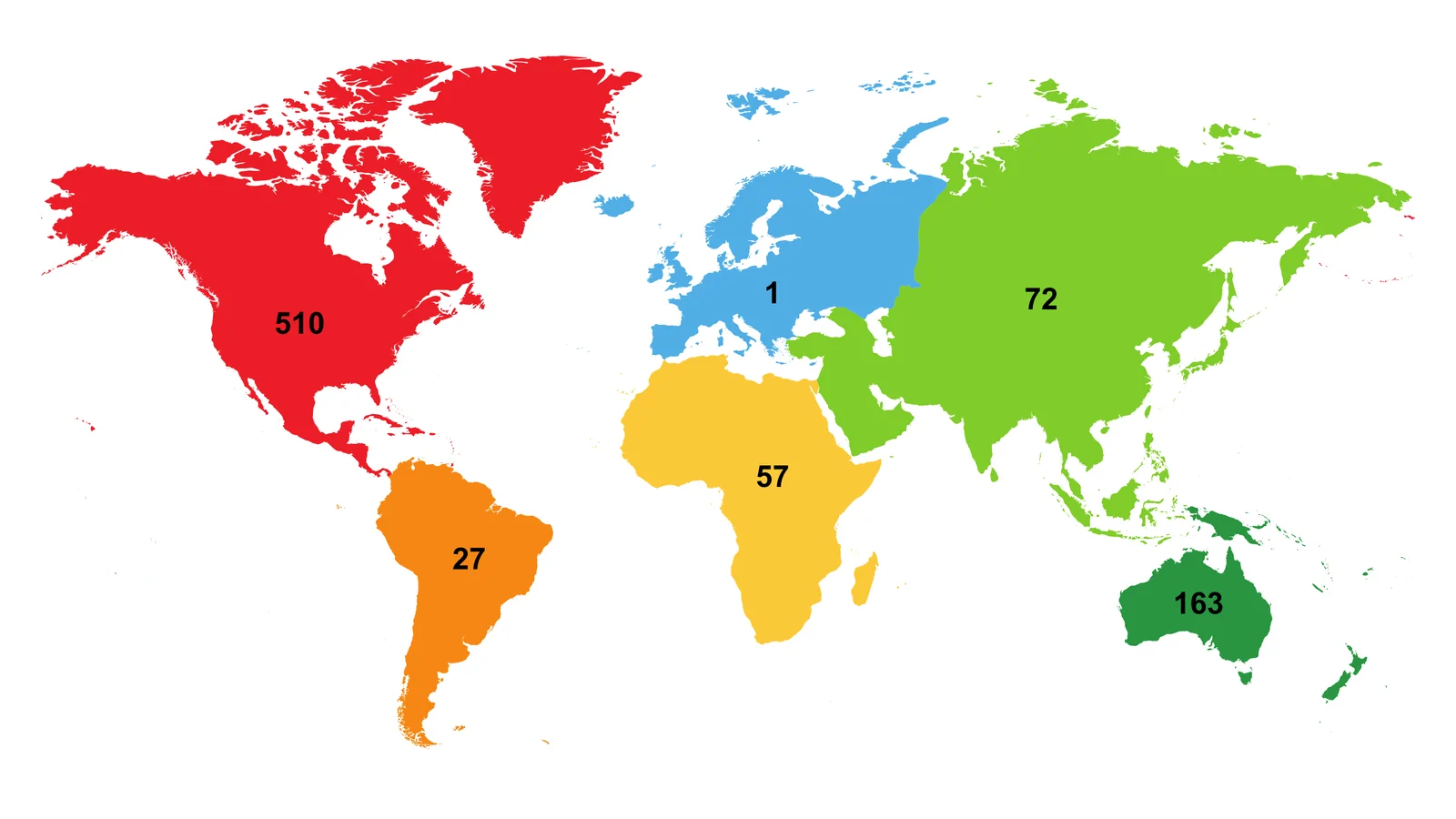
Continental origin of *Carcharhinus leucas* voucher specimens attributed with spatial metadata (n = 830).

**Figure 11.**
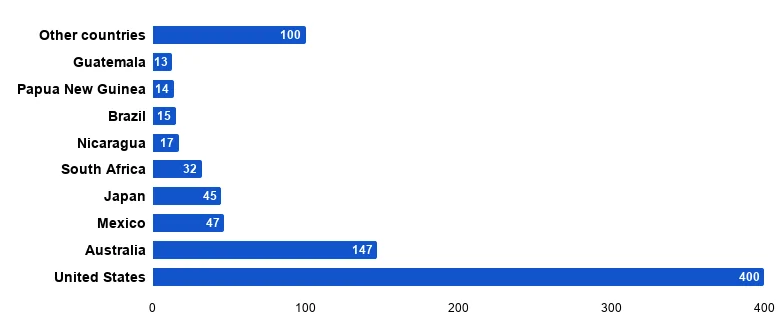
Countries where material from *Carcharhinus leucas* attributed with spatial metadata has been collected, with absolute numbers of specimens (n = 830).

Our investigation identified a total of 964 *C. leucas* voucher specimens housed across 88 institutions and museums globally (Figs 8, 9). Of these, 830 specimens possessed locality descriptions sufficient for georeferencing (Figs 10, 11), while 134 vouchers lacked geographic data and were therefore excluded from the spatial analysis. The highest number of *C. leucas* voucher specimens is housed in the collection of the Florida Museum of Natural History (UF) (n = 242), followed by the Commonwealth Scientific and Industrial Research Organisation (CSIRO) (n = 82), and the Smithsonian Institution, National Museum of Natural History (NMNH) (n = 82) (Fig. 9). Only twenty two institutions stored 10 or more *C. leucas* vouchers. Of these, only two — the South African Institute for Aquatic Biodiversity (SAIAB) and the Universidad Nacional Autónoma de México (UNAM) — are located in the Global South, whereas the remaining twenty institutions are concentrated in the Global North. This distribution illustrates the disparity in the global availability of museum specimens and reflects the greater financial resources available to institutions in the Global North for the long-term curation of scientific material.

Analysis of the continental origin of the *C. leucas* specimens revealed that the majority (n = 510) derived from the North American continent (including Central America), followed by the Australian continent (including Oceania) (n = 163). Significantly fewer specimens originated from the remaining continents: Asia (n = 72), Africa (n = 57), South America (n = 27), and Europe (n = 1) (Fig. 10).

Regarding the country of origin, most specimens were collected in the United States (n = 400), followed by Australia (n = 147), Mexico (n = 47), and Japan (n = 45) (Fig. 11). Fifteen countries were represented in the total count by only a single voucher specimen: Bangladesh, Belize, Borneo, China, Dominican Republic, French Guiana, Gabon, Haiti, Jamaica, Portugal (Azores Archipelago), Saint Lucia, The Seychelles, Somalia, United Kingdom (Bermuda), and Yemen.

**Figure 12.**
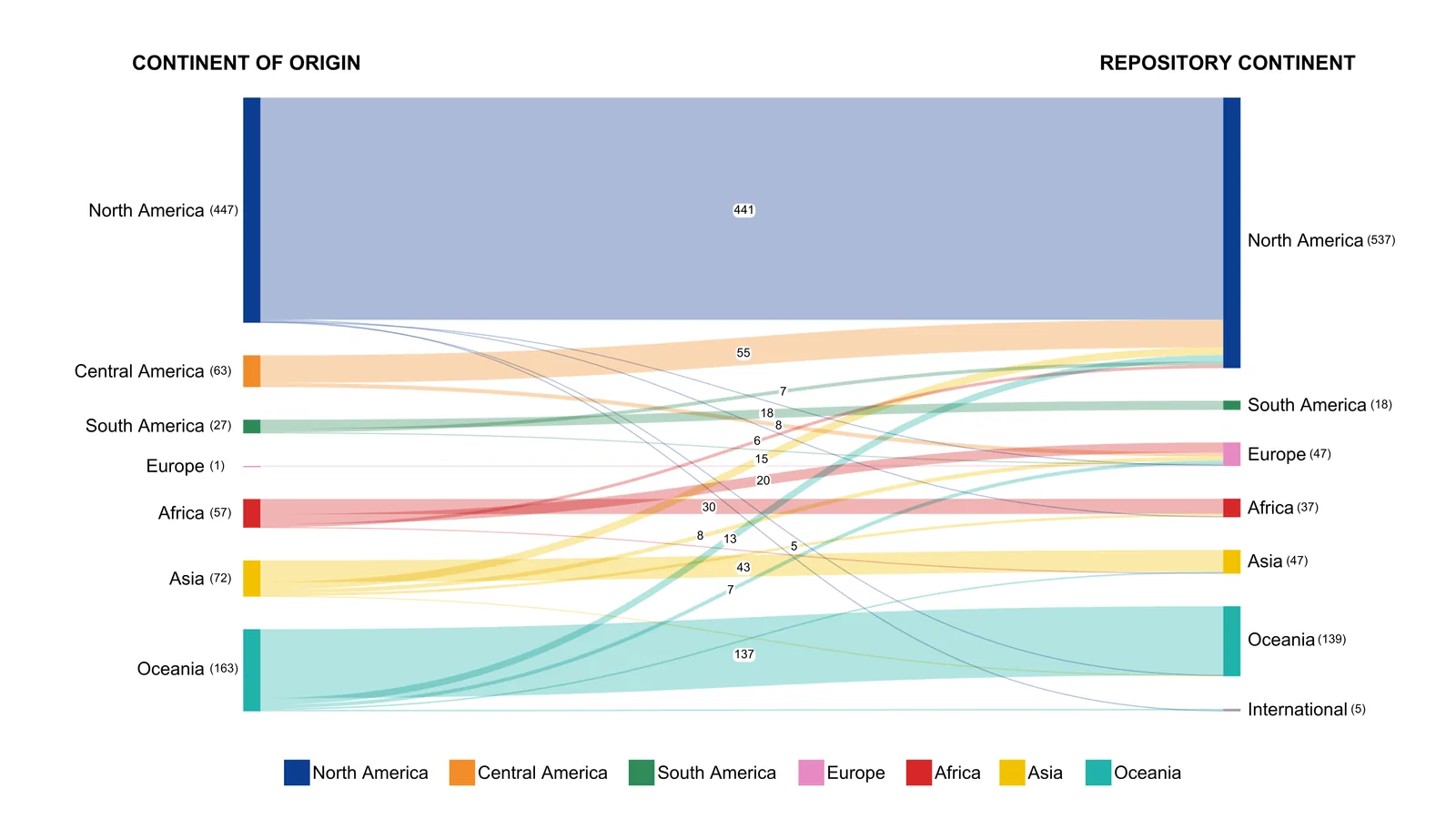
Sankey diagram showing the flow of specimens from their continent of origin to the continent where the repository institution is located. The width of each flow is proportional to the number of records (n = 830). Numbers in parentheses indicate the total number of records associated with each continent.

Analysis of the 830 georeferenced specimens revealed distinct patterns of distribution flows across different geographic scales. At the continental level, 715 specimens (86.1%) are retained within the continent where the individual was collected, whereas 115 (13.9%) were transferred to institutions on other continents (Fig. 12). At the country scale, 635 specimens (76.5%) are maintained within the country of collection, while 195 (23.5%) were transferred to foreign institutions (Suppl. material 7 ).

North America exhibits the highest regional retention in the dataset: of the 447 specimens collected there, 441 (98.7%) remain in domestic repositories. Furthermore, this continent stands out as the primary global repository, receiving specimens from all other continents. The United States of America (USA), in particular, emerges as the focal depository for these multi-origin collections, receiving 49.2% (n = 96) of the 195 specimens deposited outside their country of origin worldwide. Thus, North America dominates the global specimen network, highlighting the prevalence of intra-regional retention. Similarly, Oceania exhibits robust regional retention, heavily influenced by Australia's repository infrastructure. Asia also displays significant regional retention, primarily driven by Japan, alongside a notable outward flow of specimens to North America (Fig. 12 , Suppl. material 7 ).

Conversely, none of the specimens collected in Central America (n = 63) were retained within their region of origin. Instead, all records were deposited in external institutions, specifically in North American (n = 55, 87.3%) and in European (n = 8, 12.7%) repositories. Africa and South America exhibit moderate retention patterns. Africa retained 52.6% of its collected specimens locally, with the remainder distributed externally, predominantly in Europe, while South America retained 66.7%, accompanied by specimen transfers directed primarily toward North American repositories. Finally, "International" repositories appeared exclusively as a low-volume destination, represented by five voucher specimens deposited at the Save Our Seas Foundation (SOSF) (Suppl. material 1), a non-profit international organization not managed by a specific nation.

Therefore, our analysis revealed that the voucher specimens of *C. leucas* are predominantly deposited in institutions located in the Global North, especially in the USA, but also in European countries such as England, France, and Sweden (Fig. 8, Suppl. material 7). Miqueleiz et al. (2025) remarked on the unfortunate circumstance that the deposition of type specimens being historically deposited in Northern Hemisphere collections, a legacy of scientific collecting activities in the 19th and 20th centuries, hinders biodiversity research in the Global South, the region from which much of this material derived. Regarding specimen custody, Mills and Lassiter (2026) recently noted significant discrepancies between housing locations and original collection sites. This geographic mismatch presents a logistical barrier to research, necessitating international travel or the physical transport of vouchers, the latter of which represents a considerable risk of material damage. Consequently, scientists from the Global South face substantial barriers to accessing material collected within their own borders, a situation that perpetuates scientific inequity.

Moreover, we found that the collected material of *C. leucas* was referenced in 43 scientific publications (Suppl. material 2). This highlights the critical scientific value of voucher specimens for fish inventories and elasmobranch taxonomy. For instance, Garrick (1982) examined 55 *C. leucas* vouchers to inform his comprehensive revision of the genus *Carcharhinus*. Apparently, these specimens served as the primary basis for the species characterization of *C. leucas* by this author.

However, we were unable to include voucher specimens from certain collections due to unsuccessful communication with curators or the lack of availability in online repositories. This data gap includes material from the following collections: FMM, MPEG, MOVI, NUPEC, and UERJ. Future access to these collections may further refine the taxonomic and distributional assessment of the species addressed herein.

Information regarding specific shark specimens in ichthyological collections remains limited, as only a few studies have addressed this topic so far. De Maddalena (2006) presented a survey of 101 *Carcharodon carcharias* specimens housed in 42 different European museum institutions, assisting in the historical reconstruction of this iconic species in the Mediterranean Sea. Mollen and Iglésias (2023) compiled a summary of *Echinorhinus brucus* (Bonaterre, 1788) specimens housed in natural history collections worldwide for an assessment of its conservation status. Despite the utility of such data, comparable studies on elasmobranchs remain scarce. However, Long et al. (2011) demonstrated that museum specimens are essential for defining the geographic range of sharks and filling spatial data gaps.

Regarding the bull shark, one fundamental problem is that information regarding *C. leucas* vouchers is fragmented across numerous collections and databases worldwide, and this also applies to any other species of shark. This requires researchers to expend significant effort to aggregate dispersed data. In the present study, we collated all this information into a single synthetic overview, including an analysis of the geographic coverage of the specimen's origin. Our synthesis revealed that the *C. leucas* collected material is useful for answering some important research questions regarding distribution, historic freshwater habitat use, and the verification of records in contested localities (e.g., Samoa), as well as identifying crucial habitats such as nurseries (e.g., the Ganga-Brahmaputra-Meghna estuary system). All these findings showcase the scientific value of museum collections.

However, only a few specimens have been digitized; so continued digitization efforts by the curators are essential to enhance accessibility. Moreover, digitizing collections can mitigate the effects of catastrophic events, such as the 2018 fire at the National Museum of Brazil (UFRJ), which resulted in the irretrievable loss of vast portions of the collection (Serejo et al. 2026). As such, digitizing vouchers ensure the long-term survival of the information contained in museum collections as well as preserving the museological heritage (Silva 2019). In this context, Buckner et al. (2021) urged researchers generating new genome assemblies to deposit voucher specimens in accessible, permanent collections and link them to publications and public databases.

Historically, museum collections have served as rich sources of information for research. With a focus on voucher specimens of elasmobranchs, research in the past centred on topics such as parasite infestation (e.g., Kawanishi and Ohashi 2020). Nevertheless, given recent technological advances — including imaging technologies and molecular analyses — the potential for studying biological specimens and linking information across disciplines has expanded significantly (Kapun et al. 2025). Prasetyo et al. (2024) recently highlighted the prospects of museomics, which allows for the examination of genomic data, such as ancient (aDNA) and historic (hDNA) DNA, from shark museum material. Holmes et al. (2025) demonstrated that even stable isotope values can be derived from preserved elasmobranch jaws without damage from preserving fluids, enabling the determination of foraging habitats and trophic positions.

Focusing on *C. leucas*, a cosmopolitan species with unresolved taxonomy, there is a clear need to identify distinct lineages and populations to improve our understanding of population structure and refine management units for conservation. Therefore, scientific examination of *C. leucas* museum specimens using state-of-the-art methods can contribute to elucidating the taxonomic complexity surrounding this species and filling existing knowledge gaps regarding its biogeography. Voucher material collected from various sites across the species' range could provide a foundation for scientific studies targeting the taxonomic re-evaluation and revision of this putative species complex, which likely consists of several cryptic but distinct taxa (Naylor et al. 2012). The examination of museum material of *C. leucas* could contribute to unveiling the hidden diversity within this species.

## Conclusions, future perspectives, and recommendations

From a holistic approach, comprehensive databases encompassing voucher specimens across the full geographical range and diverse genotypes are valuable tools for a better understanding of the general biology of cosmopolitan shark species, including *C. leucas*. However, our study revealed how far we are from reaching this goal, as producing such overviews currently requires substantial effort.

As information on *C. leucas* voucher specimens is dispersed across numerous databases and museum information systems worldwide, some existing records were not incorporated in our survey, as previously noted. Therefore, our comprehensive study doesn't claim completeness. Nevertheless, the results presented here identify several regions within the cosmopolitan distribution of *C. leucas* that remain underrepresented in ichthyological collections. Notably, material is lacking for wide stretches of coastline in specific ocean basins, as well as for numerous freshwater locations where the species has been documented.

When utilizing data from public repositories, it should be noted that such records must always be treated with caution, since some entries may lack expert verification. A notable example for this issue is a record of *C. leucas* assigned to Easter Island (Chile) in the South-Central Pacific Ocean in both the iDigBio and GBIF databases (iDigBio – Integrated Digitized Biocollections 2026, GBIF – Global Biodiversity Information Facility 2026). Because one of the authors (PG) questioned the occurrence of *C. leucas* on this remote oceanic island, he requested photographic material of the voucher specimen (CMNFI-1968-1860.1) from the fish collection of the Canadian Museum of Nature (Gausmann 2021). Upon revision, the specimen was identified as *Carcharhinus galapagensis* based on the presence of an interdorsal ridge — a feature absent in *C. leucas* — and further diagnostic characteristics. This correction represented the first scientific record of the Galapagos shark for Easter Island (Gausmann 2026). Therefore, it is advisable to subject ambiguous information to a plausibility check and, whenever possible, to verify the physical material.

A temporal analysis of the voucher specimens reveals that the majority of the material was collected during the second half of the 19th century and the first half of the 20th century (Suppl. material 1). Collecting sharks in the field and their subsequent deposition in natural history collections are currently not very popular in shark science, partly due to the relatively high effort required for the preservation process, and ethical considerations associated with working with dead animals. Shark scientists, who are predominantly conservationists, refrain from killing sharks, even for scientific purposes due to animal ethics. However, the killing of specimens for this purpose is not mandatory when material is obtained from incidental bycatch, entanglements in nets of anti-shark control programs, or occasional shark strandings. Expanding collected material, especially from the geographic gaps identified herein, is scientifically desirable and could help resolve various research questions concerning *C. leucas*.

Consequently, we recommend collecting specimens of *C. leucas* in the field when the opportunity arises. Samples should be preserved immediately in fixative or preservative solutions, and associated metadata should be meticulously recorded, including the precise geographic coordinates, date, collector(s), and environmental parameters (e.g., water temperature, weather conditions). Incomplete metadata and taxonomic inaccuracies undermine the confidence and certainty of elasmobranchs collected material (Fearing et al. 2025), hindering scientific progress. Therefore, maintaining robust metadata associated with vouchers is paramount. For long-term storage in wet collections, it is advisable to use highly concentrated ethanol (96%-100%) as preservation solution instead of formalin to facilitate subsequent genomic analyses, since ethanol does not damage DNA (Schiller et al. 2014). We recommend collecting entire juvenile specimens as well as jaws from large adults, as both can provide critical diagnostic features. Furthermore, material should ideally be undamaged and properly deposited in appropriate scientific collections with unique catalog numbers to ensure accessibility, following the recommendations by Harrison et al. (2011).

The long-term curation of elasmobranch specimens can be challenging and requires certain standardized preservation protocols. Common limitations in specimen conservation include DNA fragmentation within tissue over time — especially resulting from inadequate cold storage and alcohol evaporation — alongside physical degradation caused by shrinkage, desiccation, and improper handling of delicate structures. Moreover, the toxicity and deleterious effects of fixatives such as formaldehyde, which was extensively used in the past, requires a systematic transition to safer alternatives, like ethanol, which is currently recommended.

In the context of dark diversity and from a global perspective, numerous studies have unveiled morphological differences (Bass et al. 1973, Garrick 1982) and population structuring (Karl et al. 2011, Testerman 2014, Pirog et al. 2019, Glaus et al. 2020, Laurrabaquio‐Alvarado et al. 2021, Devloo‐Delva et al. 2023, Postaire et al. 2024) among *C. leucas* populations across different ocean basins. These findings led Naylor et al. (2012) to hypothesize that *C. leucas* represents a species complex consisting of several distinct taxa. In the event of a future taxonomic revision of *C. leucas*, existing vouchers housed in natural history collections would be essential as potential type specimens for new taxonomic assessments and descriptions. As such, representative material collected reflecting the hidden genetic diversity of this species is valuable for a novel taxonomic treatment of the cosmopolitan *C. leucas*.

Since many large-bodied carcharhinids exhibit circumglobal distributions and significant population structuring, a worldwide sampling of *C. leucas* specimens is essential for achieving a comprehensive representation of genotypes in museum collections. Moreover, the philopatric behavior of *C. leucas* promotes small-scale distributed genetic homogeneity, resulting in distinct populations within specific river basins (Devloo‐Delva et al. 2023). Given that geographic isolation drives genetic diversification by limiting gene flow, as seen in distinct island populations recognized in *C. leucas* (Glaus et al. 2020), only a global investigation of museum specimens can elucidate the hidden diversity and regional genomic differences in bull sharks. Postaire et al. (2024) remarked on the scarcity of available genetic data for the species, a limitation that could be resolved through extensive acquisition of voucher specimens and associated tissue sampling for genetic databases.

Furthermore, we recommend prioritizing the collection of *C. leucas* material from the inland waters of Southeast Asia and Northern Australia. In these regions, other sympatric euryhaline carcharhinids occur that are morphologically similar to *C. leucas* (e.g., *Glyphis* spp., *Scoliodon laticaudus*), increasing the risk of misidentification. Such targeted sampling would allow for definitive verification of shark records in these freshwater environments. Historically, records of *C. leucas* have masked occurrences of river sharks (*Glyphis* spp.), compromising the accuracy of scientific datasets. Obtaining material from localities with multi-species occurrences will mitigate taxonomic confusion regarding sympatric sharks in non-marine environments (e.g., White and Last 2012, De Silva 2014). Finally, we propose the collection of specimens from marine environments where *C. leucas* and its sibling counterpart, *C. amboinensis*, are sympatric, to more accurately define their respective distributional ranges.

Museum collections contribute significantly to science through the long-term curation of specimens collected in the field, ensuring their availability for subsequent taxonomic, genetic, and morphological investigations. Their value, especially for taxonomy and systematics, is paramount, as they provide and preserve vouchers of taxonomic importance, such as type specimens (e.g., holotypes, syntypes, paratypes). In this context, Turney et al. (2015) emphasized that scientific findings must be verifiable and grounded in repeatability, a requirement achieved through specimen-level research and the deposition of labeled, curated, and digitized voucher specimens. Such records facilitate verification of the work and ensure taxonomic consistency across the research community.

Given the declining financial support for science and museum institutions, numerous scientific collections face an unsecure future. Lack of financial support poses a significant threat to their persistence, as budgetary cuts undermine the dual objectives of maintaining natural history collections and documenting biodiversity. To preserve biological heritage, specimens from at-risk or deactivated collections should be transferred to other institutions with the capacity to incorporate and maintain them. In this context, Vendramel et al. (2025) highlighted the challenges of maintaining specimens in optimal conditions, noting that scientific collections are susceptible to deterioration, institutional instability, and major disasters, all of which can result in the loss of material and associated records.

Currently, biodiversity loss and species extinction are occurring at a faster rate than taxonomic classification (Costello et al. 2013). Notably, sharks and rays are the second most threatened group of vertebrates globally, after amphibians (Jabado et al. 2025). As elasmobranch taxonomy and systematics remain understudied, the development of a worldwide museum collection database that includes material of *C. leucas* obtained from its entire range is a desirable target to facilitate access for researchers addressing questions of taxonomy, distribution, and conservation. In this context, Rocha et al. (2014) emphasized that in times of increasing rates of habitat loss and global change, responsibly collecting voucher specimens and associated metadata, and openly sharing this knowledge in public databases (e.g., GBIF, iDigBio, and VertNet), is more necessary today than ever before. Therefore, we recommend the expansion of natural history museum collections by continuing field sampling of sharks for scientific purposes, with a specific emphasis on *C. leucas*.

## Supplementary Material

D798D70F-73A6-58D8-8228-DB9F8A3BFD96 10.3897/BDJ.14.e205769.suppl1 Supplementary material 1 Synoptic table Data type Voucher specimens of *Carcharhinus leucas* File: oo_1691795.xlsx https://binary.pensoft.net/file/1691795 Mariana Haueisen, Luiz Fernando Salvador-Jr., Peter Gausmann

3B1D8730-97FA-52C9-8FF6-CBAAB85AAF1A 10.3897/BDJ.14.e205769.suppl2 Supplementary material 2 Literature references Data type Literature references for voucher specimens of *Carcharhinus leucas* and habitat classification File: oo_1772317.xlsx https://binary.pensoft.net/file/1772317 Mariana Haueisen, Luiz Fernando Salvador-Jr., Peter Gausmann

4B92D3B9-D7C4-52CB-9DBA-E1FCA02BA01C 10.3897/BDJ.14.e205769.suppl3 Supplementary material 3 Institutional Acronyms Data type Institutional Acronyms list File: oo_1691797.xlsx https://binary.pensoft.net/file/1691797 Mariana Haueisen, Luiz Fernando Salvador-Jr., Peter Gausmann

F854DA0F-4EC8-54A6-93C9-403E12360D3A 10.3897/BDJ.14.e205769.suppl4 Supplementary material 4 Detailed maps Data type Maps File: oo_1691798.pdf https://binary.pensoft.net/file/1691798 Mariana Haueisen, Luiz Fernando Salvador-Jr., Peter Gausmann

DD131F4E-1AAE-5A3E-9B91-AFFB35BE2602 10.3897/BDJ.14.e205769.suppl5 Supplementary material 5 Habitat classification and associated voucher specimens Data type Habitat classification File: oo_1691799.xlsx https://binary.pensoft.net/file/1691799 Mariana Haueisen, Luiz Fernando Salvador-Jr., Peter Gausmann

C841F3F5-397C-51A1-8092-3D9ACC5EF7B2 10.3897/BDJ.14.e205769.suppl6 Supplementary material 6 Notable specimens of *Carcharhinus leucas*
Data type Text information Brief description Extended information on scientifically significant *Carcharhinus leucas* specimens. File: oo_1824651.pdf https://binary.pensoft.net/file/1824651 Mariana Haueisen, Luiz Fernando Salvador-Jr., Peter Gausmann

3839EC1F-DF03-50AE-89E7-18CC6E6642B4 10.3897/BDJ.14.e205769.suppl7 Supplementary material 7 Sankey diagram for countries Data type Diagram File: oo_1772357.pdf https://binary.pensoft.net/file/1772357 Mariana Haueisen, Luiz Fernando Salvador-Jr., Peter Gausmann
