# Supplementary material for: Global review of bull shark Carcharhinus leucas (Valenciennes, 1839) museum specimens: summary of records, geographic analysis, and assessment of their scientific value
Source: Biodivers Data J. 2026 Nov 2;14:e205769. doi: 10.3897/BDJ.14.e205769 (PMC13749067; doi:10.3897/BDJ.14.e205769)
Supplement: Supplementary file 1 [file bdj-14-e205769-s004.pdf]

Supplementary Material 4: Geographic distribution of *Carcharhinus leucas* voucher specimens (detailed maps)

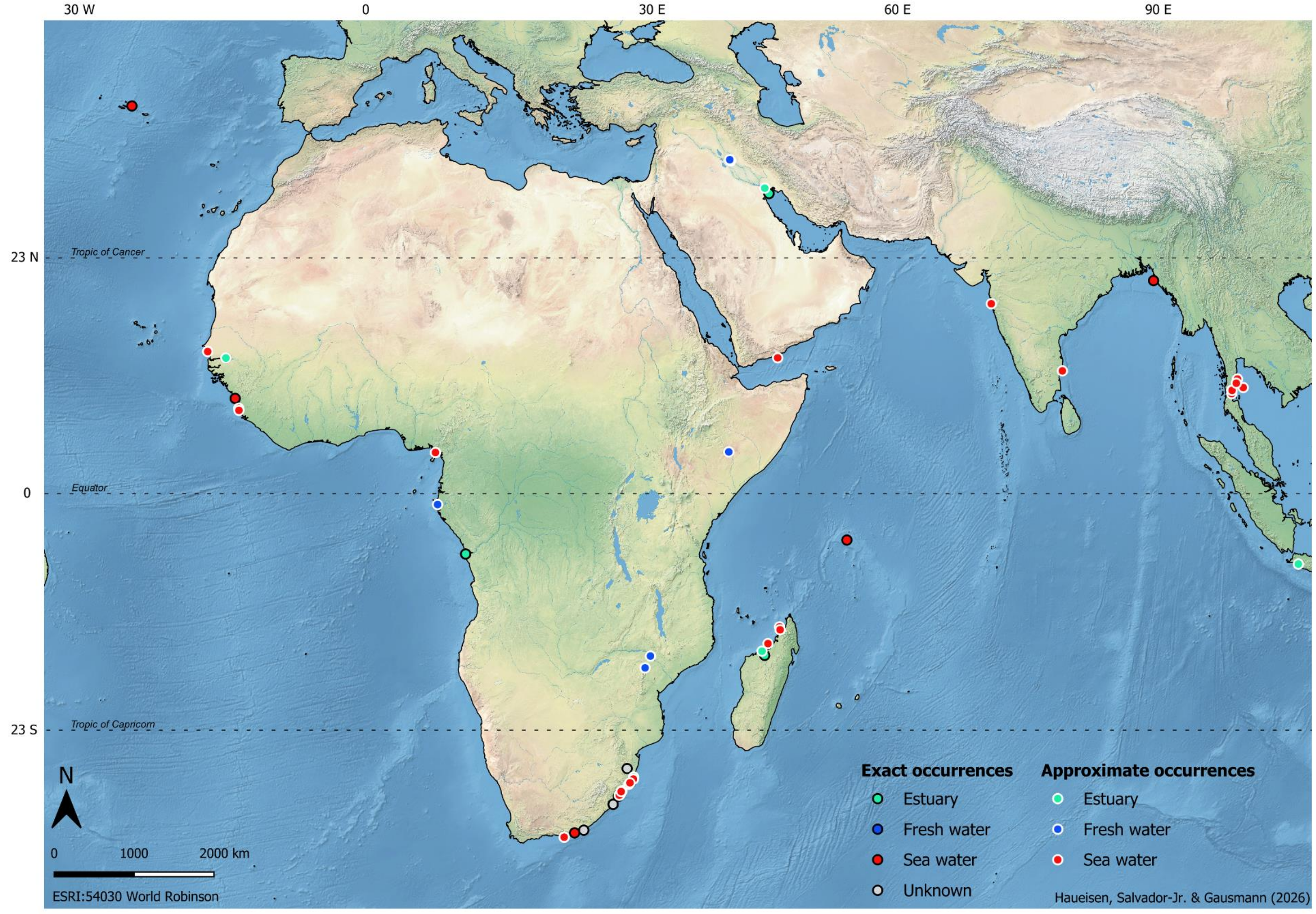

Geographic origin of *Carcharhinus leucas* vouchers collected across the European, African, and Asian continents (Eastern Atlantic, and Western and Central Indian Oceans).

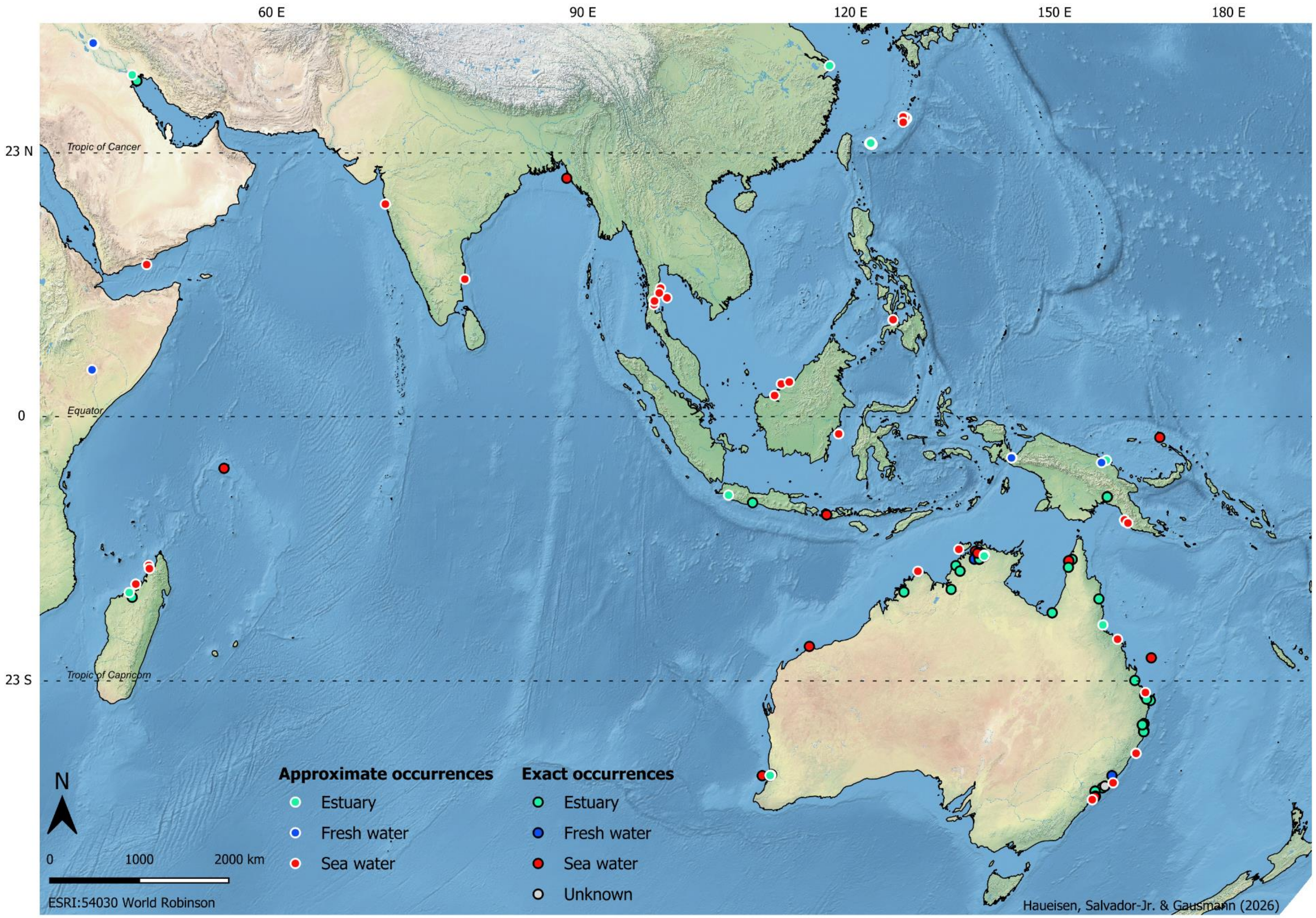

Geographic origin of *Carcharhinus leucas* vouchers collected across the Asian and Oceania continents (Indian and Western Pacific Oceans).

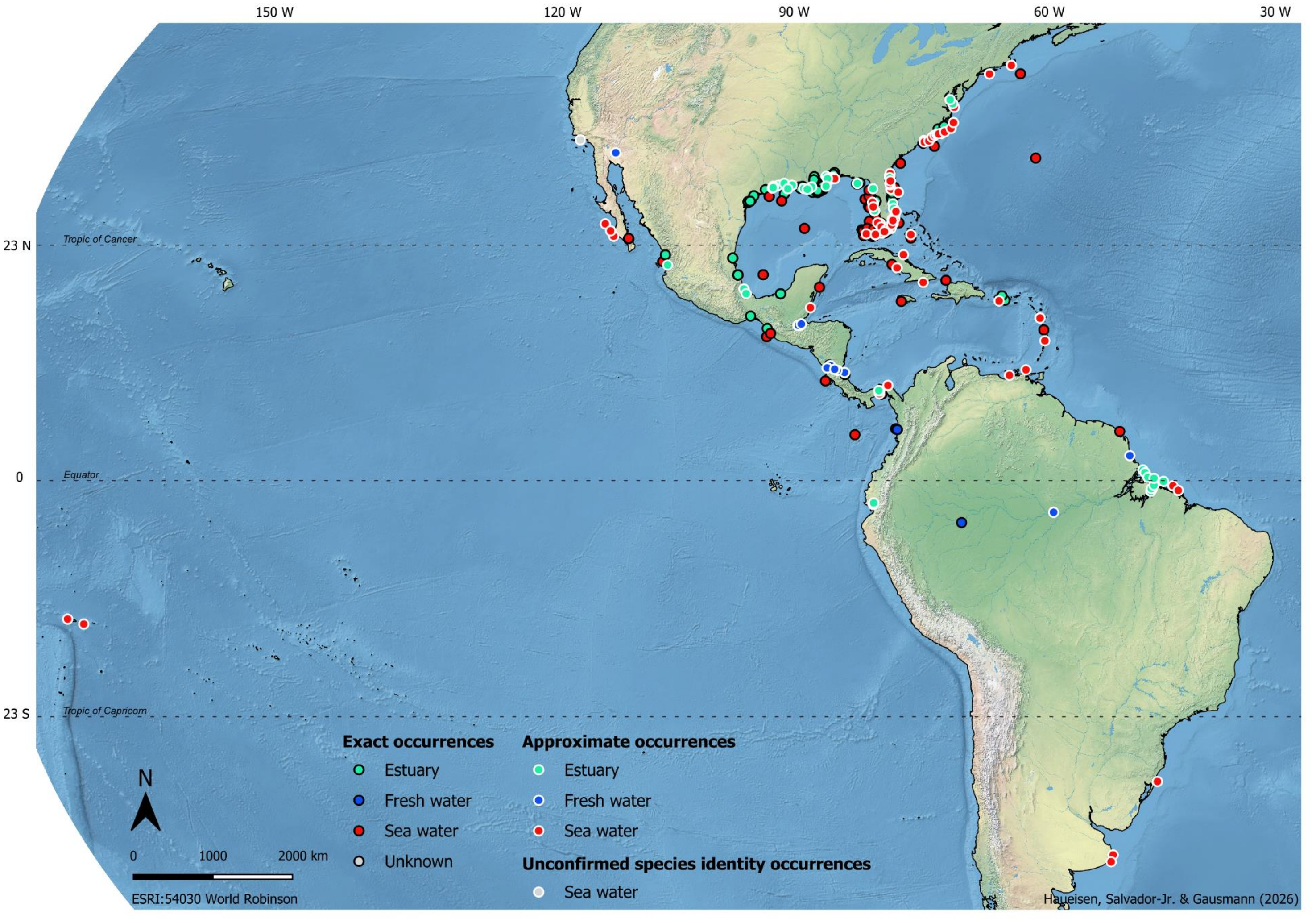

Geographic origin of *Carcharhinus leucas* vouchers collected across the North, Central, and South American continents (Western Atlantic and Eastern Pacific Oceans).
