# Supplementary material for: Global review of bull shark Carcharhinus leucas (Valenciennes, 1839) museum specimens: summary of records, geographic analysis, and assessment of their scientific value
Source: Biodivers Data J. 2026 Nov 2;14:e205769. doi: 10.3897/BDJ.14.e205769 (PMC13749067; doi:10.3897/BDJ.14.e205769)
Supplement: Supplementary file 2 [file bdj-14-e205769-s006.pdf]

Supplementary Material 6: Extended information on scientifically significant *Carcharhinus leucas* specimens.

1. MNHN-IC-A-9650 – 160 cm TL, adult, ♂ and MNHN-IC-A-9652 – 186.9 cm TL, ♀, both from the Antilles

The original species description of the cosmopolitan *Carcharhinus leucas* is based on specimens from the Caribbean Sea, consequently representing the Atlantic Ocean region. Four type specimens were collected from the Antilles — the species' designated type locality — and deposited in the Muséum national d'Histoire naturelle (MNHN) in Paris (Gray 1851). While two were apparently lost (presumably in the turmoil of the Second World War), two taxidermied specimens (MNHN-IC-A-9650 and MNHN-IC-A-9652) remain extant and in good condition (Fig. 3). It is assumed that the French zoologist Achille Valenciennes based his 1839 description of *Carcharias* (*Prionodon*) *leucas* (published in Müller and Henle 1841) on one of these four individuals. However, Garrick (1982) questioned this attribution, noting significant discrepancies between the measurements of these specimens and the dimensions provided in the original 1841 description. Nevertheless, as these two individuals represent only remaining syntypes of *C. leucas*, they hold immense taxonomic and historical significance for the species.

Interestingly, Müller and Henle (1841) reported that the four original specimens were deposited as dry mounts within the Paris collection. They were collected and transferred to the MNHN by the French naturalist Auguste Plée during his 1820 expedition to the Antilles, which included visits to islands such as Martinique, Guadeloupe, and Puerto Rico. Despite these historical records, the precise collection localities for these specimens remain unknown, with their provenance broadly documented only as the Antilles.

2. ISZZ-4468 – 76 cm TL, ♂, Zambezi River at Tete, Mozambique

A specimen of *Carcharhinus leucas* (ISZZ-4468, 76 cm TL, ♂; Fig. 4A) of special biogeographical importance was collected in 1845 by the German naturalist Wilhelm Carl Hartwig Peters and remains extant in the ichthyological collection of the Museum für Naturkunde Berlin, Ichthyologische Sammlung. This record originates from the Zambezi River system at Tete (East Africa), representing a freshwater occurrence situated approximately 418 km inland from the coast (Peters 1852). Its outstanding importance for natural history derives from the fact that this specimen represents the first verified record of *C. leucas* from a strictly freshwater environment worldwide, constituting the earliest documented occurrence of the species in a non-marine habitat since its formal description by Valenciennes in 1839 (Müller and Henle 1841).

Peters (1852) described this juvenile specimen from the Zambezi River as *Carcharias* (*Prionodon*) *zambezensis* Peters, 1852 (now recognized as a synonym of *C. leucas*), designating it as the holotype. Notably, in the original description, Peters acknowledged the strong morphological affinity between this specimen and *C. leucas*. Later, Peters (1868) produced a comprehensive description of *Carcharias zambezensis*, providing detailed morphometrics for the male juvenile and further characterization of the type locality. In this work, he also documented an additional occurrence of the species at the town of Sena, also in Mozambique, situated along the same Zambezi River (Peters 1868; Paepke and Schmidt

1988). Moreover, Peters (1868) recognized the pronouncedly blunt snout of the specimen — a diagnostic feature characteristic of *C. leucas* — and emphasized that the presence of this species in freshwater is remarkable as well as the morphological resemblance to *C. leucas* from the Antilles. From this point forward, the scientific community became increasingly aware that the presence of sharks in freshwater was not merely an anecdotal curiosity but a widespread phenomenon (Gausmann 2021).

More than a century later, Garrick (1982: 83) revised this specimen as *C. leucas*, stating: “This specimen, still in the Berlin Museum, agrees with *leucas* in all respects.” In fact, this specimen represents the juvenile stage of the euryhaline *C. leucas* and reflects the ontogenetic strategy of this species to utilize freshwater in its early life stages. While it is now understood that immature *C. leucas* spend years in low-salinity environments to reduce mortality from marine predators (Heupel and Simpfendorfer 2011), this ecological function and the mechanisms behind primarily marine taxa entering freshwater were unknown at the time.

3. NMW-61418 – 66 cm TL, ♂, Amazon River at Belém, Pará State, Brazil

The authors paid special attention to a voucher specimen of *Carcharhinus leucas* (NMW-61418) collected in 1903 along the Amazon River at Belém (Pará State, Brazil) by the Austrian ichthyologist Franz Steindachner during an expedition to Brazil (Böhme 1996). It was investigated by one of the authors (PG) in the ichthyological collection of the Naturhistorisches Museum Wien (NMW) and consists of a well-preserved male neonate (66 cm TL), with all diagnostic features clearly visible (Fig. 4B).

However, this specimen was originally labeled by Steindachner as “*Carcharhinus platyodon* Poey 1860”, an old synonym of *C. leucas* commonly applied to this species at that time in Central and North America. Although Steindachner never mentioned this specimen in any of his publications, it was subsequently referenced by other researchers.

In 1963, the specimen was examined by shark taxonomist John Garrick and reidentified as *C. leucas* (Anja Palandačić, pers. comm.). Subsequently, Garrick cited it in his comprehensive revision of the genus *Carcharhinus* Blainville, 1816 (Garrick 1982). Nevertheless, Garrick was focusing on features of anatomical and systematic significance and apparently overlooked the spatiotemporal context of this record within the large Amazon estuary, approximately 130 km from the Atlantic Ocean.

Previous to our finding, a 73 cm TL specimen of *Carcharhinus platyodon* (= *C. leucas*) (AMNH-4031), collected by the American ichthyologist Edwin Starks in 1911, was considered the earliest record of *C. leucas* in the Amazon (Starks 1913; Thorson 1972; Gausmann 2021). Although it likely originated from the vicinity of Belém within the vast Amazon estuary, this specimen was purchased at a market in the city and its exact capture location remains unclear. Consequently, it was excluded from our distributional map due to the lack of precise geolocation data.

As Steindachner’s 1903 specimen (NMW-61418) predates Starks’ 1911 record (AMNH-4031), the first stands as the earliest scientific evidence of *C. leucas* in the Brazilian Amazon, holding significant biogeographic and historical value. However, collectively, both records provide evidence of repeated habitat use within Brazilian brackish waters, which presumably serves as an important nursery area for the species (Feitosa and Nunes 2020;

Gausmann et al. 2025). Nonetheless, information on habitat use and residency duration within the world's largest river system is still scarce.

Moreover, our review of historical literature on the ichthyofauna of Brazil revealed a significant and surprising finding: Steindachner's specimen from Belém represents not only the first historical record for the inland brackish waters of Brazil, but simultaneously the first confirmed occurrence of this species for the entire country. Soto (2001) compiled an extensive bibliography for Brazilian sharks and referred to the historical works of Boulenger (1897) and Goeldi (1898) as references for the presence of *C. leucas* in Brazil. Werder and Alhanati (1981) also referred to Boulenger (1897) as a source for the occurrence of *C. leucas* in the Amazonian basin. However, our critical re-examination of Boulenger (1897) and Goeldi (1898) historical references revealed that they do not contain information about *C. leucas*. Instead, both authors referred to *Carcharias porosus* Ranzani, 1839, which is considered to be a synonym of *Carcharhinus porosus* (Ranzani, 1839) — the Smalltail Shark. In the Western Atlantic Ocean this small size carcharhinid is distributed from the northern Gulf of Mexico to southern Brazil, living near muddy bottom in the shallow continental shelf and estuaries (Ebert et al. 2021). This distinction between these two simpatric species in the coastal waters of the Western Tropical Atlantic Ocean was also noted by Thorson (1972) and Myers (1952), who recognized Boulenger's record as *C. porosus* without attributing it to *C. leucas*.

While it remains possible that both Boulenger and Goeldi intended to report *C. leucas* but were unable to achieve an accurate taxonomic identification, leading to a misidentification as *C. porosus,* their reports lack supporting vouchers specimens or illustrations. Therefore, there is no reason to doubt their records of *C. porosus*. In conclusion, given the lack of earlier valid records, the specimen housed in the Naturhistorisches Museum Wien (NMW-61418) turned out to be the first scientific record of the bull shark for Brazil.

4. NHMUK-ZOO-1874.4.28.9 (BMNH-1874.4.28.9) and NHMUK-ZOO-1924.10.1.1 (BMNH-1924.10.1.1), Tigris River near Bagdad, Iraq

Historical records of *Carcharhinus leucas* within the Tigris River drainage are represented by two notable juvenile specimens collected near Baghdad, approximately 850 km inland from the Persian Gulf. The earliest record dates back to 1874, when a juvenile specimen (~76.0 cm TL, NHMUK-ZOO-1874.4.28.9, formerly BMNH-1874.4.28.9) was collected by William Colvill and deposited in the British Museum by F. Sharpey (Günther 1874). Although Günther (1874) initially recorded this voucher under the name *Carcharias gangeticus*, reflecting the taxonomic uncertainties surrounding freshwater sharks at the time, its identity was later confirmed as *C. leucas* by Leonard Compagno (Gausmann 2021). Günther noted that this specimen, which represents the first scientific record of *C. leucas* in the Tigris River, was perhaps the most significant find among this river basin ichthyofauna.

The second record was documented fifty years later, in 1924, at Al Karrada, just upstream from Baghdad. This specimen (NHMUK-ZOO-1924.10.1.1, formerly BMNH-1924.10.1.1), measuring 124.4 cm TL, was donated by the Bombay Natural History Society and is now housed in the ichthyological collection of the Natural History Museum (London). Although only the head was preserved (Fig. 4C), its taxonomic identity was verified as *C. leucas* by John Garrick (Garrick 1982). Photographs of both vouchers are currently featured in the comprehensive review of Iranian freshwater sharks by Coad (2015).

These vouchers are among the most frequently cited in scientific literature due to their collection at exceptionally inland locations (Suppl. material 2). They substantiate the historical utilization of the Tigris River's upper reaches by juvenile *C. leucas*, demonstrating that the species once undertook extensive long-distance migrations within this system. Such findings highlight the historical role of the Euphrates-Tigris-Karun-Shatt Al-Arab system (Iraq/Iran) as a critical nursery area for the species within the Arabian/Persian Gulf, a region otherwise characterized by a paucity of suitable nursery habitats for *C. leucas* (Moore 2018).

Presently, however, the upper Tigris River stretches where both specimens were collected are no longer accessible to migratory fish due to the construction of anthropogenic barriers downstream Baghdad (Coad 2015). These habitat modifications, derived from human infrastructure development, have significantly altered the river's connectivity, likely rendering large parts of the system inaccessible or degraded for bull sharks. Unfortunately, the lack of recent data on habitat use in this system, compounded by protracted political and armed conflicts in the region, continues to preclude modern field research on the status of *C. leucas* within this river system.

5. ZSI-10250 – 65 cm TL, newborn, ♂, Hooghly River, India

Both Compagno (1984, 1988) and Talwar (1991) cited a voucher specimen of *Carcharhinus leucas* (ZSI-10250; ♂, 65.0 cm TL, neonate), currently deposited in the Zoological Survey of India. The specimen was collected from India's Hooghly River in 1867 by John Anderson and its identity was validated by Compagno (1984), providing concrete evidence of the species' historical presence in the system. Although we did not examine the voucher directly, we have no reason to doubt this diagnosis.

The Hooghly River, a major distributary of the Ganga River system into the Bay of Bengal, is characterized by strong tidal influence extending 295 km upstream (Rose et al. 2015), maintaining a pronounced salinity gradient ranging from brackish to freshwater. Due to the lack of precise locality data for the 1867 voucher, it remains unclear whether it was collected in brackish or strictly freshwater reaches, in both of which *C. leucas* has been documented. For instance, Das et al. (2025) recently reported a single specimen of *C. leucas* (83.0 cm TL, 4.5 kg) caught incidentally in a fishing net near Chandni Ghat, located 180 km upstream from the river mouth, where water conditions were nearly limnetic (0.19 % salinity). Hence, to maintain the spatial accuracy of our analysis, the ZSI-10250 record was omitted from the current distributional map.

Altogether, the presence of this historical neonate, combined with the recent record of an immature specimen (Das et al. 2025), suggests persistent, long-term habitat use of the Hooghly River by *C. leucas*. This indicates that the numerous branches of the Ganga-Brahmaputra-Meghna estuary may serve as a nursery ground for *C. leucas* population in the Bay of Bengal, a region where this species has recently been reported to occur in low abundance (Haque et al. 2021), potentially resulting from unsustainable fishing pressure.

Moreover, this 1867 voucher specimen occurrence is particularly significant given that the Hooghly River is the type locality of *G. gangeticus* where it occurs along with *C. leucas* (Compagno 1984). Therefore, the validation of such a record is crucial due to the persistent taxonomic controversy involving both species with sympatric distribution in Southeast Asia. Their shared preference for low-salinity habitats — particularly in the

Ganga-Brahmaputra-Meghna River system, including their numerous tidal-influenced branches of the Sunderbans (India/Bangladesh) (Compagno 1988; Compagno 1997; Roberts 2006; White and Last 2012; Chowdhury et al. 2017; Gausmann 2021) — and morphological similarity have led many authors to question their occurrence in strictly freshwater environments (Roberts 2006), countering previous claims of their presence throughout the system (Compagno 1988; Talwar 1991). To address this, Compagno (1988) even devoted a special chapter to untangling the status of freshwater sharks in the Hooghly River, which highlights the complexity of this topic. Because both euryhaline shark species have been frequently misidentified, Compagno (1997) noted that most historical records and specimens originally labelled as *G. gangeticus* actually represent *C. leucas*. White and Last (2012) further emphasized the significant conservation implications arising from such misidentifications, noting that distinguishing between these two carcharhinids can be challenging even for experts. This diagnostic difficulty underscores the immense value of verified museum specimens for ensuring taxonomic accuracy.

In conclusion, the preserved Hooghly specimen substantiates the species' historical presence in the vast Ganges-Brahmaputra-Meghna delta and provides irrefutable evidence of its estuarine habitat use alongside the sympatric *G. gangeticus*. Thus, this juvenile male voucher elucidates the distribution of sharks in Indian rivers and helps resolve the longstanding ambiguity surrounding euryhaline elasmobranchs in Southeast Asian riverine habitats through a verified record that can neither be neglected nor disputed.

6. CAS-ICH-55483 – Preserved specimen, jaws only, Samoa

Wass (1984) first reported *Carcharhinus leucas* in the literature for the Polynesian island of Samoa. In fact, there is a preserved specimen from this locality (CAS-ICH-55483, jaws only, collection date unknown), housed in the ichthyological collection of the California Academy of Sciences, was positively identified as *C. leucas* by Leonard Compagno in 1980. While it remains unclear if Wass (1984) referred to this specific specimen in his checklist, Nunis et al. (2024) recently questioned the presence of *C. leucas* in Samoa, stating that records of this species for the region require further verification. Apparently, these authors were not aware of the collected specimen from Samoa, and that it truly represents *C. leucas*. Our critical re-examination of the Samoan voucher (Fig. 5) concurs with Compagno's original diagnosis, as the tooth morphology is diagnostic of *C. leucas*.

The morphologically similar species *Carcharhinus amboinensis* was excluded following a comparative analysis of dental characters using high-resolution imagery and established literature (Bass et al. 1973; Naylor and Marcus 1994; Šanda and De Maddalena 2004; De Maddalena and Della Rovere 2005; Ng and Chen 2025). Although both species possess similar broad, serrated upper teeth, they differ significantly in lower jaw dentition (Garrick 1982). In *C. amboinensis*, the lower teeth are more slender and pointed, with a relatively broad base, whereas the tooth base in *C. leucas* is significantly narrower (see illustrations in Naylor and Marcus 1994, Šanda and De Maddalena 2004, and Ng and Chen 2025). Furthermore, the lower teeth of *C. leucas* are narrow and erect — except for the slightly oblique three or four lateralmost series — with margins that are concave to notched basally and slightly convex distally, with serrations finer than those of upper teeth (Garrick 1982). These dental characters provide high discriminating power and, as such, a helpful tool for distinguishing between these two congeners (for a direct comparison of the lower teeth

characteristics of both species, see also the brilliant plates provided in Bass et al. 1973, pp. 158-159).

These divergent morphologies of the lower tooth likely reflect distinct trophic niches. Adult *C. leucas* are dietary generalists consuming large prey items, including marine mammals (Moyer et al. 2021; Cullen and Marshall 2024), with a dentition designed for cutting large chunks of flesh (Goodman et al. 2022). Conversely, *C. amboinensis* is more specialized, feeding predominantly on fish, crustaceans, mollusks, and cephalopods (Tillett et al. 2014; Ebert et al. 2021).

Additionally, tooth counts serve as a definitive diagnostic tool: *C. amboinensis* typically possesses 11 teeth per side in the lower jaw, while *C. leucas* usually exhibits 12 or 13 (Garrick 1982). The Samoan specimen possesses 13 teeth per side (Fig. 5), definitively ruling out *C. amboinensis* and confirming the occurrence of *C. leucas* in Samoa. Therefore, the repeated examination of the collected material from Samoa by us not only agreed with Compagno's first diagnosis, but it also allows for a confirmation of the Samoan *C. leucas* record.

7. JPB (João Pedro Barreiros – private collection; uncatalogued) - jaw from a specimen from the Azores Archipelago, Portugal, estimated 250-270 cm TL, ♀

In March 1993, Azorean fishers caught a carcharhinid shark off the south coast of São Mateus, Terceira Island, whose length was initially reported by the fishers as 420 cm TL (Santos et al. 1997). This specimen was identified as an adult female of *Carcharhinus leucas*, and photos of the head were deposited at the Department of Oceanography and Fishes of the University of the Azores, Portugal (Santos et al. 1997). The jaws of this shark (Fig. 6) were preserved and stored in a private collection (JPB, uncatalogued). This caught specimen at this remote archipelago, situated in the North-Central Atlantic Ocean, was the first record of the species for the entire Azores.

Following on the first mention of this record in compilations about marine fish species recorded for the Azores Archipelago (Arruda 1997; Santos et al. 1997), Gadig et al. (2006) reported extensively on this specimen due to its atypical occurrence and its distributional importance for shark science. These authors re-evaluated the specimen's dimensions through a careful investigation of the jaw measurements, revising the estimated size to 250–270 cm TL. This estimate is a more realistic size derived from the jaw diameter of ~28 cm (Gadig et al. 2006). Considering that the largest *C. leucas* ever recorded was 400 cm TL (McCord and Lamberth 2009), the measured jaw diameter of the Azorean specimen does not support the initial claim of 420 cm TL. Therefore, Gadig et al. (2006) noted that the size reported by the fishers was significantly exaggerated. Based on the maximum sizes for adult females of *C. leucas* in the western Atlantic Ocean observed by Thorson and Lacy (1982) (251 cm TL), the Azorean specimen was likely fully mature.

Based on this record, *C. leucas* was included in a recent checklist of chondrichthyan fishes for the Azores (Barcelos et al. 2021). However, given its unusual location, the record was met with skepticism. Compagno (2016) questioned the record and suggested it might result from a misidentification with congeneric species such as *Carcharhinus obscurus* (dusky shark) or *C. galapagensis* Snodgrass and Heller, 1905 (Galapagos shark), the latter which is known to occur around this archipelago (Brum and Azevedo 1995). However, as photographic material of the preserved jaw was kindly provided to us by the collector (JPB), we were able to

examine the voucher and verify the species identity. Our examination confirms the initial finding, and we support the species determination previously made by Santos et al. (1997) and Gadig et al. (2006) based on the shape of the teeth in the upper and lower jaws, which are consistent with the teeth characteristics of *C. leucas* (Fig. 6).

This record from the remote oceanic island group of the Azores is of outstanding biogeographic importance because the species is reported to occur predominantly along continental coasts, with only a few records from oceanic islands distant from the continental shelf, such as the Tuamotu Archipelago in French Polynesia (Compagno 1984; Ebert et al. 2021; Gausmann 2021). Although partially speculative, this rare insular record may indicate a possible interoceanic exchange between ocean subbasins and connectivity between *C. leucas* populations from the western and eastern Atlantic Ocean. The Azores could potentially serve as a stepping stone within a marine corridor that has not yet been identified. While the vast stretches of open ocean waters of the Atlantic represent a biogeographic barrier to gene flow — a hypothesis supported by genetic investigations of both western and eastern Atlantic populations (Devloo-Delva et al. 2023) — future studies based on tagging or telemetry could shed light on this distributional enigma. Such research would clarify the nature of long-distance movements of *C. leucas*, similar to those recently reported by Daly et al. (2025), thereby enhancing our understanding of a possible elasmobranch population connectivity within the Atlantic basin.

Only future investigations can reveal if large-scale movements across the Atlantic Ocean by *C. leucas* occur regularly or occasionally. However, at the moment, it remains unclear if this *C. leucas* specimen was a single stray or if it was traveling along an unknown marine megafauna corridor facilitated by the Gulf Stream. Nevertheless, this record is outstanding regarding its biogeographic importance as an insular exclave distant from known distribution of this species along the North American and African continental coasts. Moreover, this record substantiates the inclusion of *C. leucas* in compilations of European and Portuguese elasmobranchs (Bailly et al. 2001; George and Zidowitz 2006; Hanel et al. 2009; Carneiro et al. 2014, 2019), and remains, to date, the only known European record of this species (Gausmann 2021).

8. Specimens with distributional data for the Pacific Ocean north of Bahía Magdalena, southern Baja California, Mexico: CAS-SU(ICH)-40091, LACM-Fish-39310-1, MCNB-DelMoral-2015-842, MCNB-DelMoral-2015-843, SIO-47-67, SIO-54-216, TCWC-Ichthyology-15531.01

In the Tropical East Pacific (TEP), the distribution of *Carcharhinus leucas* extends from Baja California, Mexico, to northern Peru (Chirichigno 1974; Love et al. 2005; Gausmann 2021), closely aligning with the TEP (Panamanian) Faunal Region boundaries defined by Briggs (1961) and Robertson and Kramer (2009). This range is closely related to the oceanographic conditions of the region, specifically the cold California Current. Flowing from high to low latitudes, this current is hypothesized to be a major thermal barrier for tropical warm-water fishes in the Northeast Pacific (Zapata and Robertson 2007). Consequently, the northernmost distributional limit for the bull shark in this region terminates at Bahía Magdalena (~25.00° N), the largest lagoonal system on the Pacific coast of the Baja California Peninsula (Rosenblatt and Baldwin 1958; Swift et al. 1993; Castro-Garibay 2014; Gonzáles-Acosta et al. 2015; Rábago-Quiroz et al. 2024). Historical evidence for this limit includes a 31 cm TL embryo (LACM-48-11) housed at the University of California, Los

Angeles, which was collected in its vicinity and originally reported under the synonym *Carcharhinus azureus* by Rosenblatt and Baldwin (1958) (Suppl. material 1).

Given that bull shark is a warm-water species with restricted thermal tolerance and vulnerability to lethal sudden temperature drops (Matich and Heithaus 2012; Lubitz et al. 2024), its distribution is likely constrained by the cold California Current. Thus, reports of *C. leucas* north of Bahía Magdalena — particularly from Southern California (USA) — have been shown to be unsubstantiated and questionable (Gausmann 2021). However, our investigation identified seven vouchers (CAS-SU(ICH)-40091, LACM-Fish-39310-1, MCNB-DelMoral-2015-842, MCNB-DelMoral-2015-843, SIO-47-67, SIO-54-216, TCWC-Ichthyology-15531.01) with associated localities either north of or within the vicinity of this known limit.

As the species had not yet been verifiably recorded north of Bahía Magdalena, these seven specimens raise questions regarding the validity of their associated data. From a biogeographical perspective, the central question is whether these records warrant a redefinition of the distribution of *C. leucas* in the Northeast Pacific, and to what extent the species truly ranges northward in this region.

8.1. CAS-SU(ICH)-40091

Voucher specimen CAS-SU(ICH)-40091 refers to preserved teeth of a carcharhinid shark collected in 1942 in US California waters one mile off Anacapa Island, Ventura County, USA (34.00° N, 119.40° W). The specimen, measuring 310 cm TL, was captured as bycatch in fisheries operating in the Northeast Pacific Ocean targeting Pacific jack mackerel (*Trachurus symmetricus* Ayres, 1855). The voucher was originally identified by Phil Roedel and Donald Fry from the California Division of Fish and Game (now California Department of Fish and Wildlife) as *Carcharhinus azureus* Gilbert and Starks, 1904, a synonym commonly applied to *C. leucas* in the East Pacific. Due to the large size of the freshly caught specimen, only the teeth were retained for preservation. This record is currently documented in the GBIF database (Fong 2026).

Unfortunately, we were unable to examine this voucher directly, as it is apparently lost within the California Academy of Sciences' fish collection (Jon Fong, pers. communication). Due to the verified presence of other species of *Carcharhinus* in Southern California that occur in more temperate waters, such as *Carcharhinus obscurus* Lesueur, 1818 and *C. brachyurus* Günther, 1879 (Compagno 1984; Love et al. 2005), and considering their similar dental morphologies to *C. leucas*, the identity of this specimen cannot be reliably confirmed. Therefore, we assessed this record preliminary as unconfirmed (Fig. 1).

8.2. LACM-Fish-39310-1

Voucher specimen LACM-Fish-39310-1 comprises a preserved jaw and associated tooth set collected by W. Beadle. The voucher is covered in the database of the GBIF (Del Moral-Flores et al. 2022). However, our request with the curator of the Natural History Museum of Los Angeles County (LACM) revealed a lack of precise collection date and locality data, as the collector neglected to note this information at the time (Todd Clardy, pers. communication). The available provenance for this voucher is broadly described as "western

Baja California, Mexico” (Northeast Pacific Ocean), a designation that is insufficiently detailed for accurate geospatial referencing.

Nevertheless, an examination of photographic evidence (Fig. 7) supports the original species identification as *C. leucas*. The jaw exhibits 13 teeth in each lower row, a diagnostic character that rules out similar carcharhinids. However, given the vague locality information, we could not definitively confirm whether this specimen originated north of Bahía Magdalena. Consequently, the record remains biogeographically uninformative regarding the species' northern distributional limit.

8.3. MCNB-DelMoral-2015-842 and MCNB-DelMoral-2015-843

The two voucher specimens MCNB-DelMoral-2015-842 and MCNB-DelMoral-2015-843, stored in the collection of the Barcelona Museum of Natural Sciences (Museu de Ciències Naturals de Barcelona; MCNB), do not represent autonomous vouchers. Instead, they refer to specimens housed in other collections, specifically SIO-54-216 (MCNB-DelMoral-2015-842) and SIO-47-67 (MCNB-DelMoral-2015-843), both of which were originally collected in the Northeast Pacific off Baja California. These two specimens attributed with the museum abbreviation MCNB-DelMoral were included in a treatise on the Mexican chondrichthyan fauna (Del Moral-Flores et al. 2015) and subsequently integrated into the GBIF database (Del Moral-Flores et al. 2022). For that reason, as these records represent redundant data points rather than additional individuals, they do not justify a northward range extension for *C. leucas* beyond its established limit at Bahía Magdalena. These specimens are further discussed below.

8.4. SIO-47-67

This voucher comprises material collected in September 1947 by a local fisher and subsequently deposited in the Scripps Institution of Oceanography (SIO). It was prepared with the location information: “Baja California; N of San Felipe; Mar de Cortés” (Scripps Institution of Oceanography 2025a). Based on rather vague geospatial information, precise georeferencing of this specimen is impossible. Therefore, we excluded this specimen from the distributional map for specimens records.

8.5. SIO-54-216

The record SIO-54-216 (approximately 244 cm TL) represents a noteworthy record from Guadalupe Island, Mexico, documented in 1954 by Carl Hubbs and colleagues. Originally noted as “very probably *C. azureus*,” this entry is maintained as a visual observation in the Scripps Institution of Oceanography (SIO) public database (Scripps Institution of Oceanography 2025b).

The status of *C. leucas* around Guadalupe Island, located approximately 780 km to the northwest of Bahía Magdalena and known to be an important seasonal aggregation site for the great white shark (*Carcharodon carcharias* Linnaeus, 1758) (e.g., Domeier and Nasby-Lucas 2007; Le Croizier et al. 2024), is somewhat unclear. Although the literature mentions bull sharks for the island's vicinity, there is no collected material that really proves

the occurrence of this species for the locality. Gallo-Reynoso et al. (2005) documented several *C. leucas* individuals preying on pinnipeds around the island in 1993. These authors stated that, according to regional fishers, this species is rare around Guadalupe Island; and it is possible that the presence of this species in this year (1993) was fostered because the water temperature was 2°C higher than normal due to the El Niño phenomenon at that time. Subsequently, Reyes-Bonilla et al. (2010) included *C. leucas* in a checklist of fishes for Guadalupe Island. We consider the prevailing thermal regime of Guadalupe Island to be only seasonally suitable for *C. leucas*, a warm-water species typically rare in waters below 20°C (Froeschke et al. 2010). Although anomalies in sea surface temperature (SST) may promote the species' occurrence north of Bahía Magdalena, average SST at Guadalupe Island reaches or exceeds 20°C only between August and October (Reyes-Bonilla et al. 2010). Consequently, this thermal window potentially allows *C. leucas* to occur in Guadalupe Island as a transient seasonal visitor.

Nevertheless, due to the challenges of identifying requiem sharks on species level, information derived from visual observations of *Carcharhinus* species always must be treated carefully and with caution. Taxonomic misidentifications among morphologically similar species within the genus have occurred numerous times (e.g., Duffy 2016), including *C. leucas* (e.g., Brunnschweiler 2009). Due to these diagnostic challenges and the presence of resembling congeners near Guadalupe Island, e.g., *C. obscurus* (Reyes-Bonilla et al. 2010), we excluded this visual record as a confirmed occurrence. This decision is further substantiated by the absence of photographic documentation, which precludes retrospective verification of the observational record.

8.6. TCWC-Ichthyology-15531.01

This voucher comprises only preserved claspers, which are fixed in formalin and were likely already dissected by John McEachran when he was curator of the Texas A&M University ichthyological collection (Kevin Conway, pers. communication). Because claspers among *Carcharhinus* congeners are very similar in appearance and lack distinctive diagnostic features, a reliable identification of *C. leucas* based exclusively on morphometric characteristics analysis of this organ was not feasible. Although the specimen is recorded from Bahía de Los Angeles, Gulf of California (28.97° N, 113.48° W), this record was excluded from the current distribution map due to the inability to verify the species identity and, consequently, its possible atypical northern location.

8.7. Concluding remarks on voucher specimens from the Mexican Northeast Pacific

Our investigation of *C. leucas* voucher records from the Mexican Northeast Pacific (north of or in the vicinity of Bahía Magdalena) revealed that available evidence is insufficient for reliable taxonomic verification. This is due to several factors: (i) the specimen is no longer extant for identity confirmation (CAS-SU(ICH)-40091); (ii) the preserved material lacks diagnostic characters, as in the case of isolated claspers (TCWC-Ichthyology-15531.01); (iii) imprecise locality data preclude exact geospatial referencing (LACM-Fish-39310-1; SIO-47-67); (iv) records represent redundant secondary entries for specimens housed in other institutions (MCNB-DelMoral-2015-842, MCNB-DelMoral-2015-843); or (v) entries are based solely on unconfirmed visual observations (SIO-54-216). Consequently, given the unsubstantiated or inaccurate nature of these seven institutional records, we emphasize that:

a) the presence of *C. leucas* at Guadalupe Island can neither be definitively confirmed nor excluded, and b) these data do not support a northward extension of the known range limit for *C. leucas* in the Northeast Pacific beyond Bahía Magdalena.

While the distribution of *C. leucas* reaches higher latitudes within the Gulf of California, where it is sheltered from the influence of the cold California Current, the species is subject to severe thermal constraints along the open Pacific coast. Consequently, occurrences north of Bahía Magdalena on the Pacific side of Baja California remains unconfirmed, establishing this bay as the northernmost distributional limit for this species. This conclusion harmonizes with the findings of Ramirez-Amaro et al. (2013), who reported *C. leucas* only south of Bahía Magdalena. In conclusion, situated approximately 1,000 km south of California (USA), this lagoonal system really seems to represent the definitive northern range limit of *C. leucas* in the Northeast Pacific Ocean, and, by extension, the northern boundary of the TEP.

References

Arruda LM (1997) Checklist of the marine fishes of the Azores. Arquivos do Museu Bocage, Nova série 3 (2): 13-162.

Bailly N, Eschmeyer W, Froese R, Quéro J, Van der Land J, Costello M, Zavodnik D, Serrão Santos R, Porteiro F (2001) Pisces. In: Costello M, Emblow C, White R (Eds) European register of marine species: a check-list of the marine species in Europe and a bibliography of guides to their identification. Patrimoines naturels 50. Publications Scientifiques du M.N.H.N., Paris.

Barcelos LM, Azevedo JM, Barreiros JP (2021) Updated checklist of Azores Chondrichthyes (Vertebrata: Gnathostomata). Biodiversity Data Journal 9: e62813. https://doi.org/10.3897/BDJ.9.e62813

Bass A, D'Aubrey J, Kistnasamy N (1973) Sharks of the east coast of Southern Africa. Vol. I. The genus *Carcharhinus* (Carcharhinidae). Investigational Report Oceanographic Research Institute 33: 1-168.

Böhme K (1996) Briefe Franz Steindachners von der Brasilien-Expedition 1903. Annalen des Naturhistorischen Museums in Wien. Serie B für Botanik und Zoologie 98: 545-568.

Boulenger GA (1897) XXVIII On a collection of fishes from the Island of Marajo, Brazil. Journal of Natural History 20 (117): 294–299. https://doi.org/10.1080/00222939708680627

Briggs JC (1961) The East Pacific Barrier and the distribution of marine shore fishes. Evolution 15 (4): 545-554. https://doi.org/10.2307/2406322

Brum J, Azevedo J (1995) First record of the Galapagos shark *Carcharhinus galapagensis* (Snodgrass and Heller, 1905) (Carcharinidae) on the Azores. Boletim do Museu Municipal do Funchal Supplement 4: 139-143. URL: https://publications.cm-funchal.pt/jspui/bitstream/100/1015/1/Bolmmf_s04A_1995_pp139-143.pdf

Brunnschweiler JM (2009) Species record and mistaken identifications: the case of bull sharks at Chumphon Pinnacle, Thailand. Marine Biodiversity Records 2: e94. https://doi.org/10.1017/S1755267209001122

Carneiro M, Martins R, Landi M, Costa FO (2014) Updated checklist of marine fishes (Chordata: Craniata) from Portugal and the proposed extension of the Portuguese continental shelf. European Journal of Taxonomy 73: 1-73. https://doi.org/10.5852/ejt.2014.73

Carneiro M, Martins R, Reiner F, Batista I (2019) Ichthyofauna of Portugal: taxonomic diversity, common and scientific names of marine fishes. IPMA 1 : 1-376.

Castro-Garibay H (2014) Diseño de un sistema de información geográfica de elasmobranquios con valor comercial para Baja California Sur, México. Facultad de Ciencias, Universidad Nacional de Autónoma de México (UNAM) URL: https://tesiunamdocumentos.dgb.unam.mx/ptd2014/octubre/0720438/0720438_A1.pdf

Chirichigno FN (1974) Clave para identificar los peces marinos del Peru. Instituto del Mar del Peru Informe 44: 1-378.

Chowdhury GW, Akash M, Haque AB (2017) Status of the Ganges river shark *Glyphis gangeticus* (Müller and Henle, 1839. Dhaka University Journal of Biological Sciences 26 (1): 111-116. https://doi.org/10.3329/dujbs.v26i1.46355

Coad BW (2015) Review of the freshwater sharks of Iran (family Carcharhinidae. International Journal of Aquatic Biology 3 (4): 218-224. https://doi.org/10.22034/IJAB.V3I4.100

Compagno L (1984) FAO species catalogue. Vol. 4. Sharks of the world: an annotated and illustrated catalogue of shark species known to date. Part 2. Carcharhiniformes. FAO Fisheries Synopsis 125 (4): 251-655. URL: https://www.fao.org/4/ad123e/ad123e00.htm

Compagno L (1988) Sharks of the order Carcharhiniformes. Princeton University Press, Princeton.

Compagno L (1997) Threatened fishes of the world: *Glyphis gangeticus* (Müller and Henle, 1839) (Carcharhinidae. Environmental Biology of Fishes 49: 400. https://doi.org/10.1023/A:1007358922066

Compagno L (2016) Sharks. In: Carpenter K, Angelis Nd (Eds) The living marine resources of the Eastern Central Atlantic. Vol. 2: Bivalves, gastropods, hagfishes, sharks, batoid fishes and chimaeras. FAO, Rome, 1122–1336 pp.

Cullen JA, Marshall CD (2024) Scaling of bite force corresponds with ontogenetic niche shifts in coastal elasmobranchs. Frontiers in Marine Science 11: 1452984. https://doi.org/10.3389/fmars.2024.1452984

Daly R, Murray TS, Roberts MJ, Schoeman DS, Lubitz N, Barnett A, Cedras R, Bolaji DA, Brokensha GM, Le Noury PM, Forget F, Venables SK (2025) Breaking barriers: transoceanic movement by a bull shark. Ecology 106 (5): 70096. https://doi.org/10.1002/ecy.70096

Das GC, Sharma SP, Tudu S, Hussain SA (2025) Documentation of vulnerable bull shark (*Carcharhinus leucas*) occurrence in the Hoogly River, East Coast of India. Journal of Wildlife Science 2 (1): 26-33. https://doi.org/10.63033/JWLS.HVHO8202

Del Moral-Flores LF, Morrone JJ, Alcocer Durand J, Espinosa-Pérez H, Pérez-Ponce De León G (2015) Listado anotado de los tiburones, rayas y quimeras (Chondrichthyes, Elasmobranchii, Holocephali) de México. Arxius de Miscel·lània Zoològica 13: 47-163. https://doi.org/10.32800/amz.2015.13.0047

Del Moral-Flores LF, Morrone JJ, Alcocer Durand J, Espinosa-Pérez H, Pérez-Ponce De León G (2022) Lista patrón de los tiburones, rayas y quimeras (Chondrichthyes, Elasmobranchii, Holocephali) de México. Museu de Ciències Naturals de Barcelona 1.11 https://doi.org/10.15470/hrl1kv

De Maddalena A, Della Rovere G (2005) First record of the pigeye shark, *Carcharhinus amboinensis* (Müller and Henle, 1839), in the Mediterranean Sea. Annales Series Historia Naturalis 15: 209-212.

Devloo-Delva F, Burridge CP, Kyne PM, Brunnschweiler JM, Chapman DD, Charvet P, Chen X, Cliff G, Daly R, Drymon JM, Espinoza M, Fernando D, Barcia LG, Glaus K, González-Garza BI, Grant MI, Gunasekera RM, Hernandez S, Hyodo S, Jabado RW, Jaquemet S, Johnson G, Ketchum JT, Magalon H, Marthick JR, Mollen FH, Mona S, Naylor GJP, Nevill JEG, Phillips NM, Pillans RD, Postaire BD, Smoothy AF, Tachihara K, Tillet BJ, Valero-Vargas JA, Felauty P (2023). From rivers to ocean basins: The role of ocean barriers and philopatry in the genetic structuring of a cosmopolitan coastal predator. Ecology and Evolution 13 (2): e9837. https://doi.org/10.1002/ece3.9837

Domeier ML, Nasby-Lucas N (2007) Annual re-sighting of photographically identified white sharks (*Carcharodon carcharias*) at an eastern Pacific aggregation site (Guadalupe Island, Mexico). Marine Biology 150 (5): 977-984. https://doi.org/10.1007/s00227-006-0380-7

Duffy CA (2016) Misidentification of *Carcharhinus galapagensis* (Snodgrass and Heller, 1905) in the Southwest Pacific Ocean. Zootaxa 4132 (1): 97-106. https://doi.org/10.11646/zootaxa.4132.1.8

Ebert DA, Fowler SL, Dando M (2021) Sharks of the world: a complete guide. Princeton University Press, Princeton.

Feitosa LM, Nunes JL (2020) New Record of *Carcharhinus leucas* in an Amazonian river system. Boletim do Laboratório de Hidrobiologia 30 (1): 62-67. https://doi.org/10.18764/1981-6421e2020.6

Fong J (2026) CAS Ichthyology (ICH). Version 150.508. California Academy of Sciences. Occurrence dataset. https://doi.org/10.15468/efh2ib. Accessed on: 2026-1-08.

Froeschke JT, Stunz GW, Wildhaber ML (2010) Environmental influences on the occurrence of coastal sharks in estuarine waters. Marine Ecology Progress Series 407: 279-292. https://doi.org/10.3354/meps08546

Gadig OB, Juliano MF, Barreiros JP (2006) Further notes on the capture of a *Carcharhinus leucas*, in a northeastern Atlantic oceanic insular shelf, the Azores archipelago, Portugal. Arquipélago - Life and Marine Sciences 23A: 63-66.

Gallo-Reynoso JP, Figueroa-Carranza AL, Blanco-Parra MD (2005) Los tiburones de la Isla Guadalupe. In: Santos-del Prado K, Peters E (Eds) La Isla Guadalupe. Restauración y conservación. SEMARNAT/INE/CICESE/GECI/SEMAR, México DF, 143–169 pp.

Garrick JA (1982) Sharks of the genus *Carcharhinus*. NOAA Technical Report NMFS Circular 445: 1-194. URL: https://repository.library.noaa.gov/view/noaa/5775

Gausmann P (2021) Synopsis of global fresh and brackish water occurrences of the bull shark *Carcharhinus leucas* Valenciennes, 1839 (Pisces: Carcharhinidae), with comments on distribution and habitat use. Integrative Systematics 4 (1): 55-213. https://doi.org/10.18476/2021.423083

Gausmann P, Matich P, Laurrabaquio-Alvarado NS (2025) Research goals of special concern for *Carcharhinus leucas* (Carcharhiniformes: Carcharhinidae) in Latin America - biological, distributional, and conservation priorities. Neotropical Ichthyology 23 (3): e250034. https://doi.org/10.1590/1982-0224-2025-0034

George MR, Zidowitz H (2006) Checkliste der europäischen Knorpelfischarten mit wissenschaftlichen und deutschen Namen. Zeitschrift für Fischkunde 8 (1–2): 71-81.

Goeldi EA (1898) Primeira contribuição para o conhecimento dos peixes do valle do Amazonas e das Guianas. Estudos ichthyologicos dos annos 1894-1898. Boletim do Museu Paraense de História Natural e Ethnographia, Pará 2: 443-488.

Gonzáles-Acosta AF, Ruiz-Campos G, Balart EF (2015) Composition and zoogeography of fishes in mangrove ecosystems of Baja California Sur, México. In: Rodriguez RR, Gonzáles-Acosta AF, Muñiz-Salazar R (Eds) The arid mangrove forest from Baja California Peninsula. I. Nova Publishers, New York, 63–80 pp.

Goodman K, Niella Y, Bliss-Henaghan T, Harcourt R, Smoothey AF, Peddemors VM (2022) Ontogenetic changes in the tooth morphology of bull sharks (*Carcharhinus leucas*). Journal of Fish Biology 101 (4): 1033-1046. https://doi.org/10.1111/jfb.15170

Gray JE (1851) List of the Specimens of Fish in the Collection of the British Museum: Part I., Chondropterygii. Edward Newman, London, 160 pp. https://doi.org/10.5962/bhl.title.20819

Günther A (1874) A contribution to the fauna of the River Tigris. The Annals and Magazine of Natural History 4 (14): 36-38. URL: https://biostor.org/reference/20532

Hanel L, Plíštil J, Novák J (2009) Checklist of the fishes and the fish-like vertebrates on the European continent and adjacent seas. Bulletin Lampetra VI: 108-180.

Haque AB, Cavanagh RD, Seddon N (2021) Evaluating artisanal fishing of globally threatened sharks and rays in the Bay of Bengal, Bangladesh. PLOS One 16 (9): e0256146. https://doi.org/10.1371/journal.pone.0256146

Heupel MR, Simpfendorfer CA (2011) Estuarine nursery areas provide a low-mortality environment for young bull sharks *Carcharhinus leucas*. Marine Ecology Progress Series 433: 237-244. https://doi.org/10.3354/meps09191

Le Croizier G, Hoyos-Padilla M, Amezcua-Martínez F, Aquino-Baleytó M, Besnard L, Le Grand F, Le Loc'h F, Mathieu-Resuge M, Munaron JM, Ory A, Sardenne F, Schaal G, Lorrain A (2024) Can biochemical tracers reveal ontogenetic trophic shift and individual prey selection in white sharks from Guadalupe Island, Northeast Pacific? Environmental Research 262 (1): 119507. https://doi.org/10.1016/j.envres.2024.119507

Love MS, Mecklenburg CW, Mecklenburg TA, Thorsteinson LK (2005) Resource inventory of marine and estuarine fishes of the west coast and Alaska: A checklist of North Pacific and Arctic Ocean species from Baja California to the Alaska-Yukon border. U. S. Department of the Interior, U.S. Geological Survey, Biological Resources Division, Seattle, 276 pp. https://doi.org/10.3133/70179564

Lubitz N, Daly R, Smoothey AF, Vianello P, Roberts MJ, Schoeman DS, Sheaves M, Cowley PD, Dagorn L, Forget FG, Soria M, Peddemors VM, Filmalter JD, Butcher PA, Brett G, Barnett A (2024) Climate change-driven cooling can kill marine megafauna at their distributional limits. Nature Climate Change 14 (5): 526-535. https://doi.org/10.1038/s41558-024-01966-8

Matich P, Heithaus MR (2012) Effects of an extreme temperature event on the behavior and age structure of an estuarine top predator, *Carcharhinus leucas*. Marine Ecology Progress Series 447: 165-178. https://doi.org/10.3354/meps09497

McCord ME, Lamberth SJ (2009) Catching and tracking the world's largest Zambezi (bull) shark *Carcharhinus leucas* in the Breede Estuary, South Africa: the first 43 hours. African Journal of Marine Science 31 (1): 107-111. https://doi.org/10.2989/AJMS.2009.31.1.11.782

Moore AB (2018) Identification of critical habitat in a data-poor area for an endangered aquatic apex predator. Biological Conservation 220: 161-169. https://doi.org/10.1016/j.biocon.2018.02.013

Moyer JK, Brooks AM, Irschick DJ (2021) Field-based feeding performance and kinematics of bull sharks, *Carcharhinus leucas* (Carcharhiniformes: Carcharhinidae. Ichthyology and Herpetology 109 (4): 963-977. https://doi.org/10.1643/i2020153

Müller J, Henle J (1841) Systematische Beschreibung der Plagiostomen. Verlag von Veit and Comp, 200 pp. https://doi.org/10.5962/bhl.title.6906

Myers GS (1952) Sharks and sawfishes of the Amazon. Copeia 1952 (4): 268-269. https://doi.org/10.2307/1439279

Naylor GJ, Marcus LF (1994) Identifying isolated shark teeth of the genus *Carcharhinus* to species: relevance for tracking phyletic change through the fossil record. American Museum Novitates 3109: 1-53.

Ng SL, Chen LX (2025) Specimen-based record of the pigeye shark *Carcharhinus amboinensis* (Carcharhiniformes: Carcharhinidae) from Taiwan. Platax 22: 63-71. https://doi.org/10.29926/platax.202512_22.0005

Nunis T, Grant MI, Chin A (2024) Sharks and rays of the Samoan archipelago: a review of their biological diversity, social and cultural values, and conservation status. Pacific Conservation Biology 30: PC23039. https://doi.org/10.1071/PC23039

Paepke HJ, Schmidt K (1988) Kritischer Katalog der Typen der Fischsammlung des Zoologischen Museums Berlin. Teil 2: Agnatha, Chondrichthyes. Mitteilungen aus dem Zoologischen Museum in Berlin 64 (1): 155-189. https://doi.org/10.1002/mmnz.19880640108

Peters WC (1852) Hr. Peter legte einige neue Säugethiere und Flussfische aus Mossambique vor. Bericht über die zur Bekanntmachung geeigneten Verhandlungen der Königlich Preussischen Akademie der Wissenschaften zu Berlin. Aus dem Jahre 1852. Berlin, 273–276. https://www.biodiversitylibrary.org/page/11054836. Accessed on: 2025-10-05.

Peters WC (1868) Naturwissenschaftliche Reise nach Mossambique, auf Befehl seiner Majestät des Königs Friedrich Wilhelm IV in den Jahren 1842 bis 1848 ausgeführt. Zoologie IV: Flussfische. Georg Reimer, Berlin, 116 pp. URL: https://publikationen.ub.uni-frankfurt.de/opus4/frontdoor/deliver/index/docId/19797/file/Peters_Mossambique_Zoo_4.pdf

Rábago-Quiroz CH, González-Acosta AF, Ruiz-Campos G, Franco-López J, García-Borbón JA, Magaña JD (2024) Composition and zoogeography of marine-estuarine fish from a lagoon system in a temperate-tropical transition zone of the Eastern Pacific. Revista Mexicana de Biodiversidad 95: e955305.https://doi.org/10.22201/ib.20078706e.2024.95.5305

Ramirez-Amaro SR, Cartamil D, Galvan-Magaña F, Gonzalez-Barba G, Graham JB, Carrera-Fernandez M, Escobar-Sanchez O, Sosa-Nishizaki O, Rochin-Alamillo A (2013) The artisanal elasmobranch fishery of the Pacific coast of Baja California Sur, Mexico, management implications. Scientia Marina 77 (3): 473-487.https://doi.org/10.3989/scimar.03817.05A

Reyes-Bonilla H, Gonzáles-Romero S, Walther Mendoza M, Calderon-Aguilera LE, Bedolla-Guzmán YR, Ramírez-Valdez A, Olivares-Bañuelos NC (2010) Checklist and biogeography of fishes from Guadalupe Island, Western Mexico. CalCOFI Report 51: 195-209.

Robertson DR, Kramer KL (2009) Shore fishes and biogeographic subdivisions of the Tropical Eastern Pacific. Marine Ecology Progress Series 380: 1-17. https://doi.org/10.3354/meps07925

Roberts TR (2006) Rediscovery of *Glyphis gangeticus*: debunking the mythology of the supposed "Gangetic freshwater shark". The Natural History Bulletin of the Siam Society 54 (2): 261-278.

Rose L, Bhaskaran PK, Kani SP (2015) Tidal analysis and prediction for the Gangra location, Hoogly estuary in the Bay of Bengal. Current Science 109 (4): 745-758.

Rosenblatt RH, Baldwin WJ (1958) A review of the Eastern Pacific sharks of the genus *Carcharhinus*, with a redescription of *C. malpeloensis* (Fowler) and California records of *C. remotus* (Duméril). California Fish and Game 44 (2): 137-159.

Šanda R, De Maddalena A (2004) Collection of the sharks of the National Museum in Prague - Part 2. Skeletal preservations. Journal of the National Museum (Praha), Natural History Series 173: 51-58.

Santos RS, Porteiro FM, Barreiro JP (1997) Marine fishes of the Azores: annotated checklist and bibliography. Bulletin of the University of Azores Supplement 1: 1-244.

Scripps Institution of Oceanography (2025a) Marine Vertebrate Collection Database. SIO 47-67. https://sioapps.ucsd.edu/collections/mv/collection/47-_67/?cl=47-67. Accessed on: 2025-11-06.

Scripps Institution of Oceanography (2025b) Marine Vertebrate Collection Database. SIO 54-216. https://sioapps.ucsd.edu/collections/mv/collection/54-216/?cl=54-216. Accessed on: 2025-11-06.

Soto JM (2001) Annotated systematic checklist and bibliography of the coastal and oceanic fauna of Brazil. I. Sharks. Mare Magnum 1 (1): 51-120.

Starks EC (1913) The fishes of the Stanford expedition to Brazil. University Series, 12. Stanford University Publications, 1–77 pp. https://doi.org/10.5962/bhl.title.13885

Swift CC, Haglund TR, Ruiz M, Fisher RN (1993) The status and distribution of the freshwater fishes of Southern California. Bulletin of the Southern California Academy of Sciences 92 (3): 101-167.

Talwar PK (1991) Pisces. In: Mohammad, Shamim J, Zoological Survey of India (Eds) Faunal Resources of Ganga: General introduction and vertebrate fauna, Zoological Survey of India. Part 1. The Survey, Calcutta, 145 pp.

Thorson TB (1972) The status of the bull shark, *Carcharhinus leucas*, in the Amazon River. Copeia 1972 (3): 601-605. https://doi.org/10.2307/1442947

Thorson TB, Lacy Jr EJ (1982) Age, growth rate and longevity of *Carcharhinus leucas* estimated from tagging and vertebral rings. Copeia 1982 (1): 110-116.https://doi.org/10.2307/1444275

Tillett BJ, Meekan MG, Field IC (2014) Dietary overlap and partitioning among three sympatric carcharhinid sharks. Endangered Species Research 25 (3): 283-293. https://doi.org/10.3354/esr

Wass RC (1984) An annotated checklist of the fishes of Samoa. NOAA Technical Report SSRF—781: 1—43. https://repository.library.noaa.gov/view/noaa/65806/noaa_65806_DS1.pdf

Werder U, Alhanati CE (1981) Informe sobre um tubarão (*Carcharhinus leucas*), capturado no Amazonas com alguns detalhes de sua morfologia externa. Acta Amazonica 11 (1): 193-196.

White WT, Last PR (2012) A review of the taxonomy of chondrichthyan fishes: a modern perspective. Journal of Fish Biology 80 (5): 901-917. https://doi.org/10.1111/j.1095-8649.2011.03192.x

Zapata FA, Robertson DR (2007) How many species of shore fishes are there in the Tropical Eastern Pacific? Journal of Biogeography 34 (1): 38-51. https://doi.org/10.1111/j.1365-2699.2006.01586.x
