# Supplementary material for: Global review of bull shark Carcharhinus leucas (Valenciennes, 1839) museum specimens: summary of records, geographic analysis, and assessment of their scientific value
Source: Biodivers Data J. 2026 Nov 2;14:e205769. doi: 10.3897/BDJ.14.e205769 (PMC13749067; doi:10.3897/BDJ.14.e205769)

Supplementary Material 7: Sankey diagram showing the flow of specimens from their country of origin to the country where the repository institution is located. The width of each flow is proportional to the number of records (n = 830). Numbers in parentheses indicate the total number of records associated with each country.

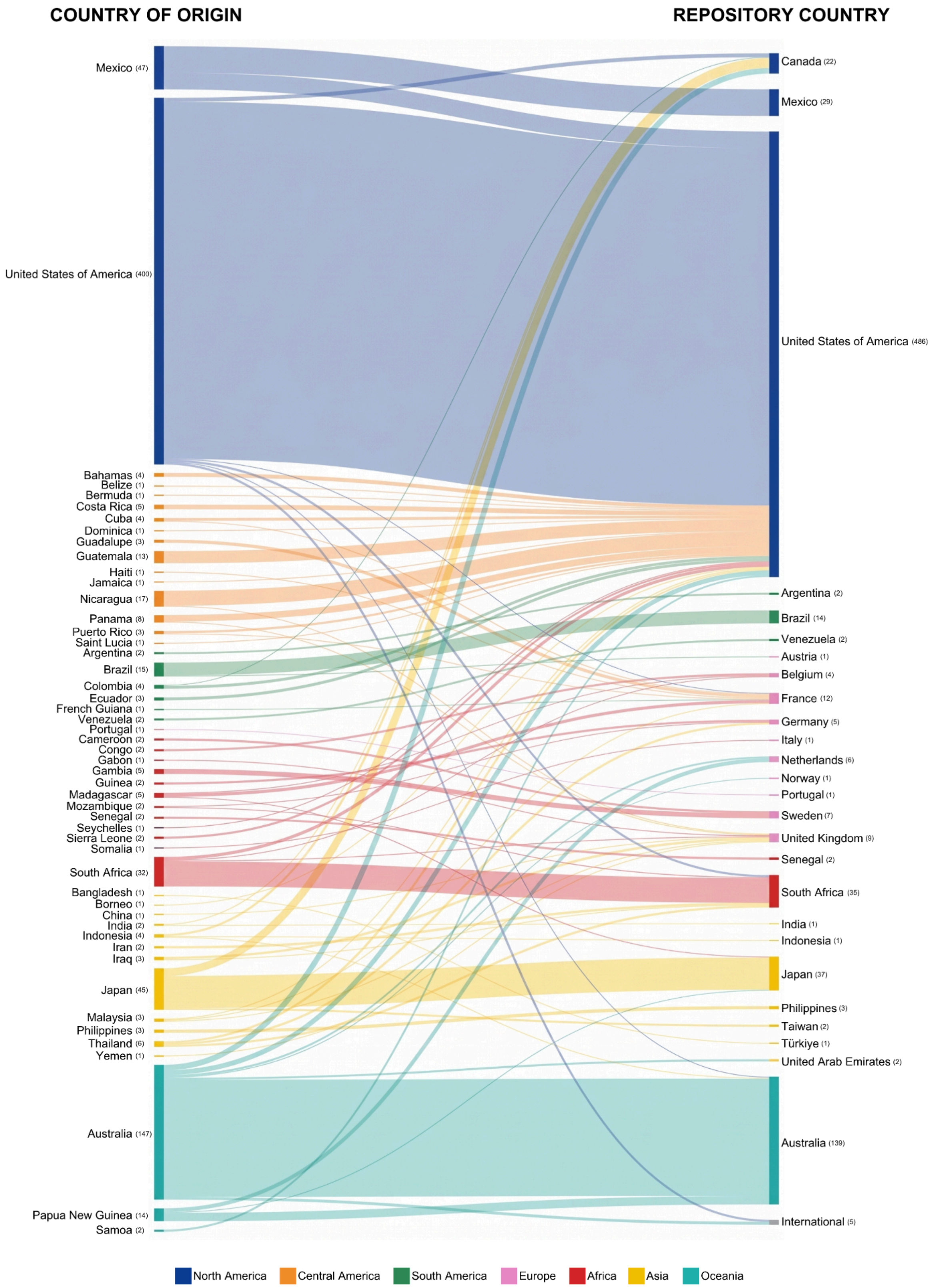

Supplement: Supplementary file 3 [file bdj-14-e205769-s007.pdf]
